# The tetrameric pheromone module SteC‐MkkB‐MpkB‐SteD regulates asexual sporulation, sclerotia formation and aflatoxin production in *Aspergillus flavus*

**DOI:** 10.1111/cmi.13192

**Published:** 2020-03-03

**Authors:** Dean Frawley, Claudio Greco, Berl Oakley, Mohamed M. Alhussain, Alastair B. Fleming, Nancy P. Keller, Özgür Bayram

**Affiliations:** ^1^ Department of Biology Maynooth University Maynooth Co. Kildare Ireland; ^2^ Department of Medical Microbiology and Immunology University of Wisconsin‐Madison Madison Wisconsin USA; ^3^ Department of Molecular Biosciences University of Kansas Lawrence Kansas USA; ^4^ Department of Microbiology, School of Genetics and Microbiology Moyne Institute of Preventive Medicine, Trinity College Dublin Dublin Ireland; ^5^ Department of Bacteriology University of Wisconsin‐Madison Madison Wisconsin USA

**Keywords:** aflatoxin B1, *Aspergillus flavus*, pheromone module, sclerotia, secondary metabolism

## Abstract

For eukaryotes like fungi to regulate biological responses to environmental stimuli, various signalling cascades are utilized, like the highly conserved mitogen‐activated protein kinase (MAPK) pathways. In the model fungus *Aspergillus nidulans*, a MAPK pathway known as the pheromone module regulates development and the production of secondary metabolites (SMs). This pathway consists five proteins, the three kinases SteC, MkkB and MpkB, the adaptor SteD and the scaffold HamE. In this study, homologs of these five pheromone module proteins have been identified in the plant and human pathogenic fungus *Aspergillus flavus*. We have shown that a tetrameric complex consisting of the three kinases and the SteD adaptor is assembled in this species. It was observed that this complex assembles in the cytoplasm and that MpkB translocates into the nucleus. Deletion of *steC*, *mkkB*, *mpkB* or *steD* results in abolishment of both asexual sporulation and sclerotia production. This complex is required for the positive regulation of aflatoxin production and negative regulation of various SMs, including leporin B and cyclopiazonic acid (CPA), likely via MpkB interactions in the nucleus. These data highlight the conservation of the pheromone module in *Aspergillus* species, signifying the importance of this pathway in regulating fungal development and secondary metabolism.

## 1 INTRODUCTION

Filamentous fungi, such as the genus *Aspergillus*, are ubiquitous in the environment and can exhibit both beneficial and detrimental effects with regard to human and plant health. The growth and development of *Aspergillus* species are often coupled with the process of secondary metabolism (Calvo, Wilson, Bok, & Keller, 2002; J. H. Yu & Keller, 2005). This results in the production of a wide range of low molecular weight, bioactive compounds known as secondary metabolites (SMs) which can exhibit a diverse array of properties (Berdy, 2005; Brakhage et al., 2009). Certain species are capable of producing SMs that are used as pharmaceuticals and antibiotics. However, other species have the ability to produce SMs such as phytotoxins, mycotoxins and carcinogens, which can lead to crop contamination, as well as infections or carcinomas in humans and animals (Bills & Gloer, 2016; Hagiwara, Sakamoto, Abe, & Gomi, 2016).

In order for *Aspergillus* species to regulate their development and secondary metabolism in response to environmental cues, various signalling pathways are utilized to detect and respond to stimuli appropriately. An example of highly conserved signalling cascades implicated in the regulation of these processes is mitogen‐activated protein kinase (MAPK) pathways (Schaeffer & Weber, 1999). These pathways consist three protein kinases, abbreviated as MAPKKK, MAPKK and MAPK, which activate one another sequentially via phosphorylation upon detection of a stimulus at a receptor. The terminal kinase (MAPK), when activated, is then capable of translocating into the nucleus where it interacts with transcription factors to regulate various biological responses (Widmann, Gibson, Jarpe, & Johnson, 1999).

The most well studied MAPK pathway in fungi is the Fus3 pheromone module in yeast (Bardwell, 2005). In this pathway, the Fus3 MAPK becomes phosphorylated in response to pheromone signalling and enters the nucleus where it activates the Ste12 transcription factor. This regulates cell–cell fusion, otherwise known as sexual development (Bardwell, 2005; van Drogen, Stucke, Jorritsma, & Peter, 2001; Wong Sak Hoi & Dumas, 2010). Since the discovery of this pathway, homologous proteins have been characterized in filamentous fungi and have been shown to regulate diverse processes such as asexual and sexual development, SM production and pathogenesis (Frawley, Karahoda, Sarikaya Bayram, & Bayram, 2018; Lev, Sharon, Hadar, Ma, & Horwitz, 1999; Li, Bobrowicz, Wilkinson, & Ebbole, 2005; Paoletti et al., 2007). A pathway that is homologous to the yeast Fus3 pheromone module has been characterized in the model ascomycete fungus *Aspergillus nidulans* (Bayram et al., 2012; Frawley et al., 2018). This pathway consists the MAPKKK SteC, the MAPKK MkkB, the MAPK MpkB, the adaptor protein SteD and the scaffold protein HamE. In response to pheromone signalling at the hyphal plasma membrane, SteC becomes anchored to the membrane by the SteD adaptor and is activated. SteC phosphorylates MkkB, which then phosphorylates the terminal kinase MpkB. The regulation of phosphorylation intensity is mediated via the scaffold protein HamE which physically interacts with both MkkB and MpkB (Frawley et al., 2018). MpkB, when activated, is then capable of translocating into the nucleus where it activates various transcription factors and regulators. One transcription factor that is activated is the Ste12 homolog SteA which regulates hyphal fusion and sexual cleistothecia development. MpkB also activates the velvet protein VeA which functions as a regulator of secondary metabolism via assembly of the velvet complex (VeA‐VelB‐LaeA) (Atoui, Bao, Kaur, Grayburn, & Calvo, 2008; Bayram et al., 2008; Bayram et al., 2012; Sarikaya Bayram et al., 2010). The *A. nidulans* pheromone module has also been shown to play roles in the regulation of vegetative growth rate and asexual sporulation. Various SMs that are regulated via this pathway include the antibiotic penicillin, the anti‐tumour compound terrequinone A and the carcinogenic mycotoxin sterigmatocystin (Bayram et al., 2012; Frawley et al., 2018).

*Aspergillus flavus* is a saprophytic fungus that is found worldwide and is considered a major global threat as it is capable of causing contamination of a wide variety of agricultural crops. Crops susceptible to infection include peanuts, corns and cereals such as maize, rice and wheat (Lewis et al., 2005; Rushing & Selim, 2019; J. Yu, Cleveland, Nierman, & Bennett, 2005). When this fungus colonizes crops, it produces a highly carcinogenic and immunosuppressive SM known as aflatoxin, along with various other SMs (Amare & Keller, 2014). When these contaminated crops are ingested, they can pose harmful effects for humans and animals, leading to the development of hepatocellular carcinomas or aflatoxicoses (Bhatnagar‐Mathur, Sunkara, Bhatnagar‐Panwar, Waliyar, & Sharma, 2015; Hedayati, Pasqualotto, Warn, Bowyer, & Denning, 2007; Kew, 2013; Liu & Wu, 2010; Williams et al., 2004). *A. flavus* exists in the soil as either vegetative hyphae, asexual conidia or sexual sclerotia (Amaike & Keller, 2011). Sclerotia are reproductive structures produced by single strains of *A. flavus* and are utilized primarily as a means of survival during harsh environmental conditions (B.W. Horn et al., 1996; D.T. Wicklow, 1987). However, via the crossing of sexually compatible strains, sclerotia can also harbour sexual ascospores for reproduction (B. W. Horn, Moore, & Carbone, 2009). *A. flavus* produces sclerotia when it infects crops (B. W. Horn et al., 2014; D.T. Wicklow, Horn, Burg, & Cole, 1984), and studies have found evidence for the co‐regulation of both sclerotia development and SM biosynthesis via similar genetic mechanisms (Calvo, 2008; Calvo et al., 2002), as well as the harbouring of many SMs, such as aflatoxins, within sclerotia (Gloer, 2007; D. T. Wicklow & Shotwell, 1983).

The correlation between development and secondary metabolism observed in both *A. nidulans* and *A. flavus* poses the question of whether *A. flavus* may also utilize MAP kinase signalling via the pheromone module to regulate its reproduction and SM biosynthesis. In this study, homologs of all five *A. nidulans* pheromone module proteins have been identified in *A. flavus*. Via a proteomic approach, we have provided evidence of the existence of a MAP kinase pheromone module in *A. flavus* consisting of the three kinases SteC, MkkB, MpkB and the adaptor protein SteD. However, HamE was not shown to interact with the members of the *A. flavus* pheromone module, unlike what is observed in *A. nidulans* (Frawley et al., 2018). This study also shows that the tetrameric complex in *A. flavus* is critical for the regulation of asexual sporulation, sexual sclerotia development and aflatoxin production. These data highlight the importance of the pheromone module as a regulator of both development and secondary metabolism in the Aspergilli.

## 2 RESULTS

### 2.1 A protein complex consisting of SteC, MkkB, MpkB and SteD exists in *A. flavus*

**Figure 1:**
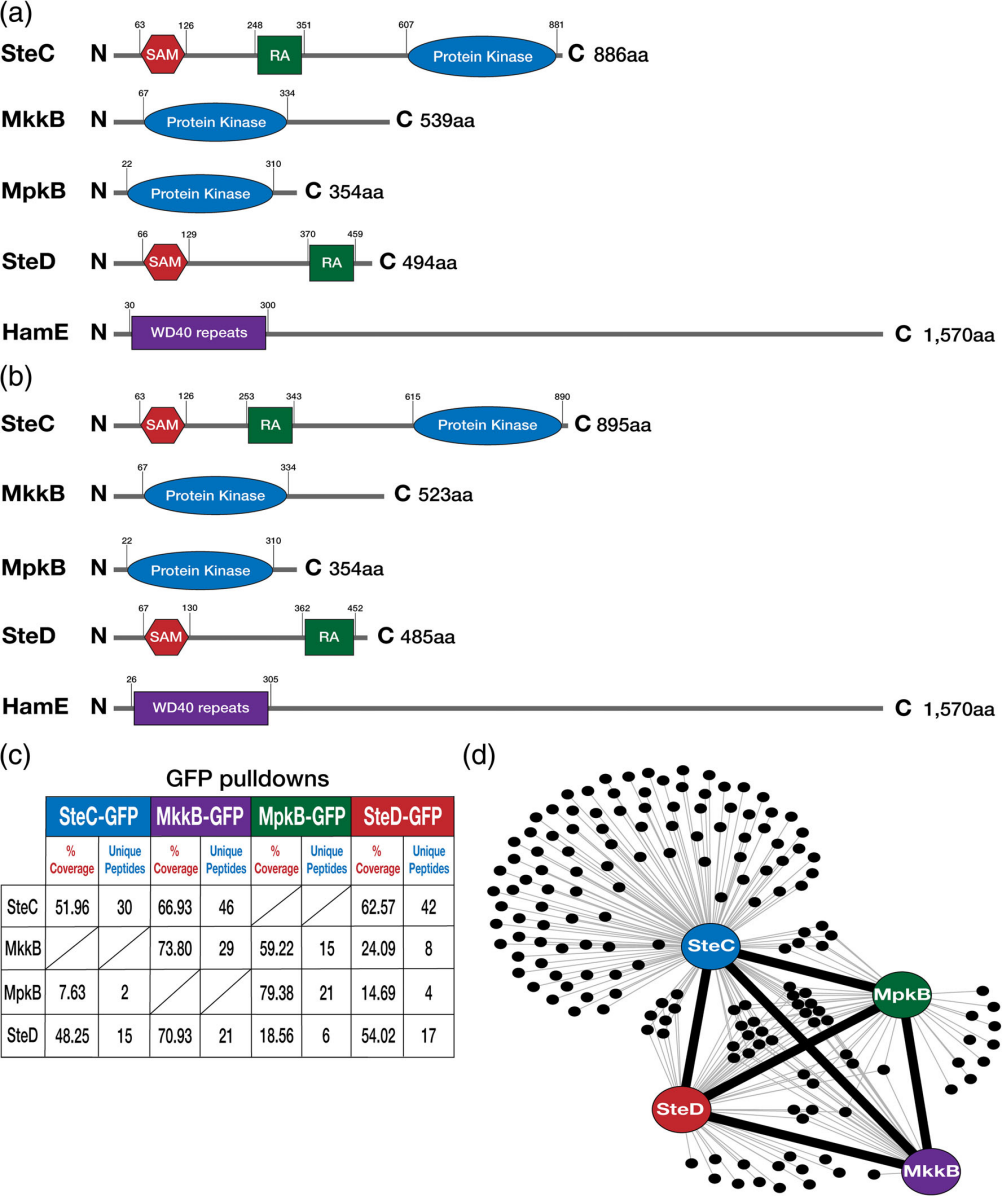
Identification of homologs of the pheromone module proteins in *Aspergillus flavus*. (a) Schematics of the pheromone module proteins in *Aspergillus nidulans*. Protein sizes are measured in amino acids (aa). N (N‐terminus), C (C‐terminus). SAM (Sterile Alpha Motif), RA (Ras‐associated). Detection of protein homologs was performed by reciprocal BLAST searches (Altschul et al., 1990). Detection of protein sizes and domains was performed using a combination of ScanProsite (de Castro et al., 2006) and InterPro software (Mitchell et al., 2019). (b) Schematics of the pheromone module protein homologs in *A. flavus*. (c) GFP‐pulldowns and LC–MS/MS analysis of the pheromone module kinases and SteD. GFP‐tagged proteins are given at the top of the table, and co‐purified proteins are given on the left side. The percentage of coverage and unique peptides of each detected protein are displayed. Two biological replicates of each strain were used. Strains were cultured vegetatively for 24 hr in complete media. (d) Interaction network of the pheromone module components based on unique peptides detected in each GFP pulldown. Each black dot represents a protein detected in two independent biological replicates but not in the wild type

In order to identify whether homologs of the pheromone module proteins exist in *A. flavus*, reciprocal BLAST searches were performed (Altschul, Gish, Miller, Myers, & Lipman, 1990). It was found that homologs of all five members of the *A. nidulans* pheromone module exist in *A. flavus*. The *A. flavus* SteC homolog (AFLA_048880) exhibits 79.25% sequence similarity, while the percentages of similarity for MkkB (AFLA_103480), MpkB (AFLA_034170), SteD (AFLA_002340) and HamE (AFLA_095770) homologs are 80.69%, 98.59%, 74.05% and 63.76%, respectively. Once these homologs were detected, “ScanProsite” (de Castro et al., 2006) and “InterPro” (Mitchell et al., 2019) software were used to determine the sizes of these proteins and the domains they possess in comparison to the *A. nidulans* proteins (Figure 1a,b). These analyses revealed that the SteC proteins in both *A. nidulans* (Figure 1a) and *A. flavus* (Figure 1b) possesses a Sterile Alpha Motif (SAM) domain near the N‐terminus between amino acids 63 and 126, as well as a Ras‐associated (RA) domain at amino acids 248–351 for *A. nidulans* and amino acids 253–343 for *A. flavus*. Both proteins also possess a protein kinase domain near the C‐terminus spanning a similar range of amino acid residues. MkkB and MpkB in both *A. nidulans* and *A. flavus* possess a protein kinase domain between the same amino acid residues. The SteD adaptor in both species contains a SAM domain near the N‐terminus and a RA domain near the C‐terminus at similar amino acid positions. Finally, the HamE protein in both species contains WD40 repeats near the N‐terminus, which are characteristic scaffolding domains (Frawley et al., 2018; Xu & Min, 2011).

To determine whether these homologous proteins form a complex in *A. flavus*, similar to that observed in *A. nidulans* (Bayram et al., 2012; Frawley et al., 2018), a synthetic green fluorescent protein (*sgfp*) epitope tag was fused to the C‐termini of the *steC*, *mkkB*, *mpkB* and *steD* genes (Figure S1). All attempts to detect the *hamE* gene tagged with *sgfp* failed, and instead, the *hamE* gene was coupled to a *3xha* epitope tag at the C‐terminus (Figure S2). The fusion proteins were immunoprecipitated from vegetative cultures grown for 24 hr and were run on a mass spectrometer to detect the tagged proteins and their interaction partners (Figure 1c). It was found that SteC‐GFP pulldowns co‐purified MpkB and SteD (Table S4), MkkB‐GFP pulldowns co‐purified SteC and SteD (Table S5), MpkB‐GFP pulldowns co‐purified MkkB and SteD (Table S6) and SteD‐GFP pulldowns co‐purified SteC, MkkB and MpkB (Table S7). Unlike in *A. nidulans* (Frawley et al., 2018), pulldowns of HamE did not result in detection of any of the pheromone module proteins (Table S8) and HamE was not detectable in any purifications of SteC, MkkB, MpkB or SteD. Together, this interactome data provide evidence of the existence of a tetrameric MAP kinase pathway consisting of SteC‐MkkB‐MpkB‐SteD in *A. flavus* (Figure 1d). Also, this data suggest that HamE may not be directly interacting with the members of this pathway.

### 2.2 The pheromone module proteins are required for the regulation of asexual sporulation

To determine whether the pheromone module proteins contribute to the regulation of asexual sporulation in *A. flavus*, the genes encoding each protein of the complex were deleted (Figures S1 and S2), and the phenotypes of the mutants were monitored. The *steC*Δ, *mkkB*Δ, *mpkB*Δ, *steD*Δ and *hamE*Δ strains were generated by replacing the gene open reading frames with either the *Aspergillus fumigatus pyrG* gene or the phleomycin resistance cassette (*phleO*), respectively. Complementation strains for each mutant were also generated by reinserting a functional copy of the gene open reading frame into the respective mutant strains.

**Figure 2:**
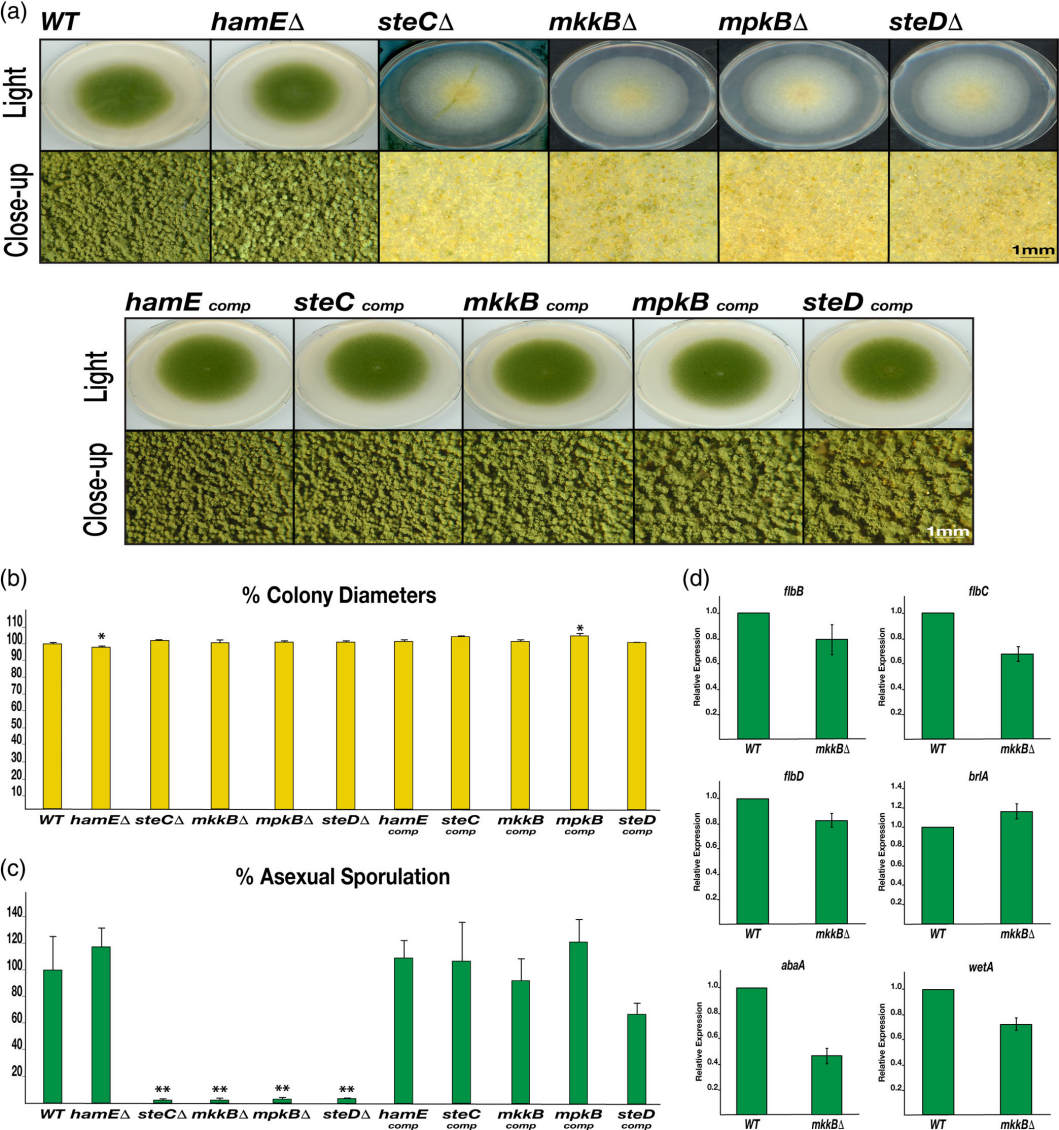
Asexual phenotypes and colony diameters of deletion and complementation strains. (a) Asexual phenotypes of each strain. The pheromone module protein deletion strains and complementation strains were spot‐inoculated (5 × 10^3^ spores) in triplicate on PDA plates containing supplements. Wild type refers to the TJES19.1 strain. These plates were incubated for 5 days in the light at 30°C to induce asexual development. Close‐up images were taken at 2× magnification using the Olympus szx16 microscope with an Olympus sc30 camera. Scale bar represents 1 mm. (b) Colony diameters of the asexually induced strains from (a). The average values from three independent biological replicates were plotted ± s.d. as a percentage of the WT strain. *p* values were calculated by performing unpaired Studentʼs *t* tests (*p < .05). (c) Quantification of asexual sporulation levels in each strain (**p < .01). (d) Relative expression levels of genes associated with the regulation of asexual development. The TJES19.1 strain and *mkkB*Δ mutant strain were inoculated (2 × 10^6^ spores) in liquid complete media and left to incubate on a shaker at 30°C for 24 hr. The mycelia were then shifted onto PDA plates and left to incubate in the presence of light for 24 hr. mRNA was isolated from two independent biological replicates per strain. Three technical replicates per biological replicate were used (N = 6). The wild type average was chosen to represent 100% expression (1.0 relative expression). *skpA* was used as a reference gene to obtain relative expression levels of corresponding genes using the 2^ΔΔCt^ method

Each mutant and complementation strain was spot inoculated on PDA plates in triplicate and incubated in the presence of light for 5 days to promote asexual conidiation (Figure 2a). It was observed that the deletion of *steC*, *mkkB*, *mpkB* or *steD* dramatically influences sporulation. Each one of these mutants exhibited a completely pale white phenotype and was unable to undergo asexual conidiation. The average percentage range of sporulation for the mutant strains was between 2.46–3.84%, in comparison to the wild‐type strain (TJES19.1) average, which was chosen to represent 100% (Figure 2c). However, the *hamE* mutant phenotype resembled the wild type and was capable of producing asexual spores. The complementation of each gene successfully restored the levels of conidiation, with each strain resembling the wild‐type phenotype. The levels of sporulation for each complementation strain were between 66.54–120.36%, with respect to the wild‐type strain. Real‐time/quantitative Polymerase Chain Reaction (qPCR) analysis was performed to assess the relative expression levels of asexual development genes in an *mkkB* mutant, in comparison to a wild‐type strain (Figure 2d). The expression of *flbC* and *abaA* genes were significantly decreased in the mutant strain, while the *flbB*, *flbD*, *brlA* and *wetA* genes did not exhibit any significant differences.

The colony diameters of each asexually induced strain were measured, and the averages of three independent replicates for each strain were plotted as a percentage of the respective wild type average (Figure 2b). Apart from the *hamE* mutant, which displayed a slightly reduced colony diameter, no significant differences were observed in the rates of vegetative growth in any of the mutants, unlike the reduced rates of growth observed in *A. nidulans* (Frawley et al., 2018). Taken together, these results indicate that the SteC, MkkB, MpkB and SteD proteins all contribute to the regulation of asexual sporulation in *A. flavus*, similar to what is observed in *A. nidulans*. However, these data suggest that these proteins are not required for the regulation of hyphal growth, and that HamE is not involved in the regulation of conidiation, unlike what is observed in *A. nidulans* (Bayram et al., 2012; Frawley et al., 2018).

### 2.3 Each pheromone module protein homologue is essential for sexual sclerotia production

**Figure 3:**
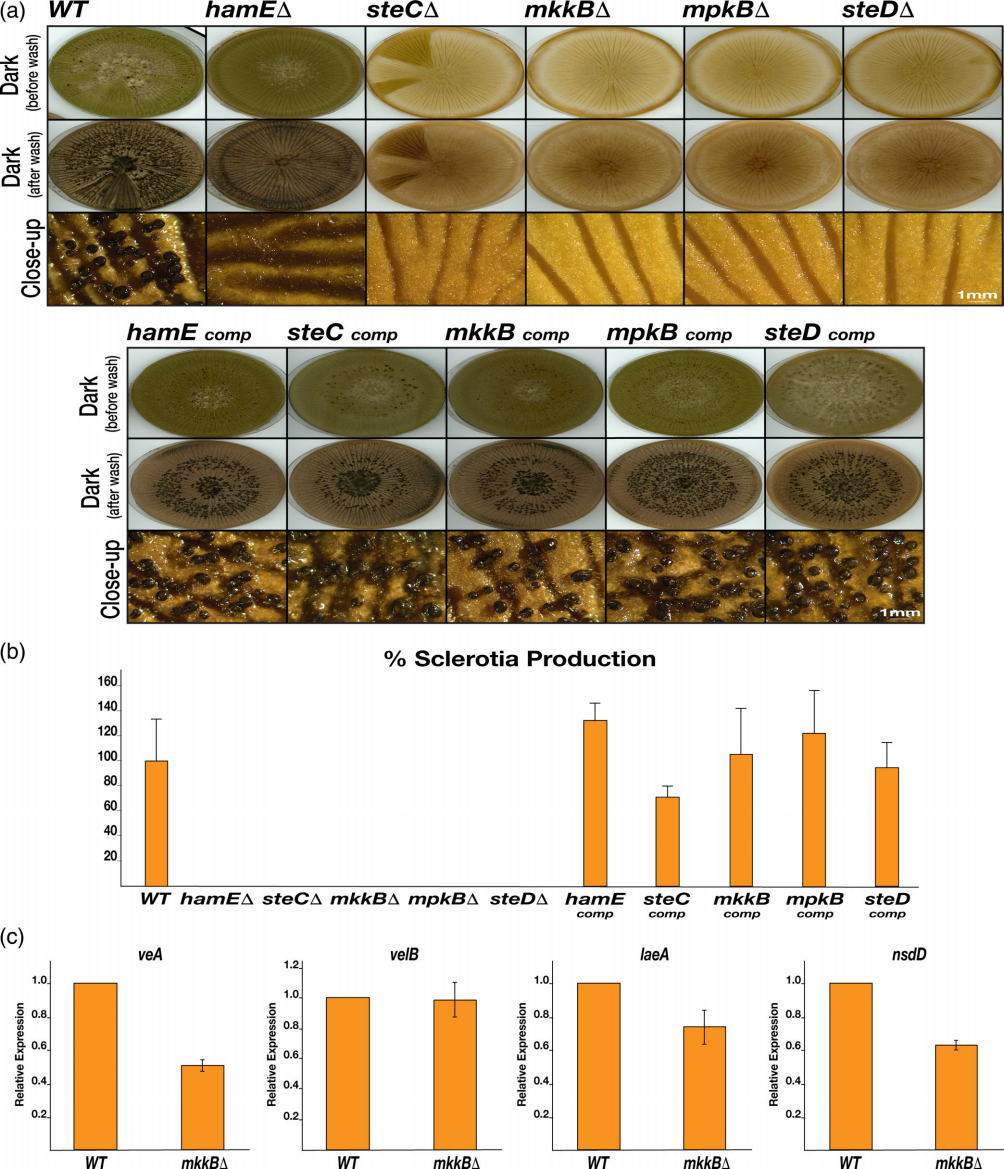
Sexual phenotypes of deletion and complementation strains. (a) Sexual phenotypes of each strain. The pheromone module protein deletion and complementation strains were spot‐inoculated (5 × 10^3^ spores) in triplicate on WHM plates containing supplements. These plates were completely covered in aluminium foil and incubated for 2 weeks in the dark at 30°C to induce sexual development. Prior to scanning, plates were washed with 70% ethanol to remove conidia and to reveal underlying sclerotia. Close‐up images were taken at 1x magnification using the Olympus szx16 microscope with an Olympus sc30 camera. Scale bar represents 1 mm. (b) Quantification of sexual sclerotia levels in each strain. The average amount of sclerotia produced by the wild‐type strain was chosen to represent 100%. Mean values of all other strains (*N* = 3) were plotted ± s.d. as a percentage of the WT. (c) Relative expression levels of genes associated with regulation of sexual development. The TJES19.1 strain and *mkkB*Δ mutant strain were inoculated (2 × 10^6^ spores) in liquid potato dextrose broth (PDB) and left to incubate on a shaker at 30°C for 72 hr. mRNA was isolated from two independent biological replicates per strain. Three technical replicates per biological replicate were used (N = 6). The average expression level values were plotted ± s.d. as a percentage of the wild type average, which was chosen to represent 100% expression (1.0 relative expression). *skpA* was used as a reference gene to obtain relative expression levels of corresponding genes using the 2^ΔΔCt^ method

The mutant and complementation strains were also tested to determine whether they play a role in the regulation of sexual development. Each strain was spot‐inoculated on Wickerham (WHM) agar plates in triplicate. These plates were completely covered in aluminium foil to generate a dark environment and strains were cultured for 2 weeks to induce sexual development and sclerotia formation (Figure 3a).

It was evident that the deletion of *steC*, *mkkB*, *mpkB* and *steD* resulted in a very clear change in phenotype, with each mutant displaying a pale white phenotype, devoid of asexual conidia. However, the *hamE* mutant displayed normal conidiation, with respect to the wild‐type strain. When each plate was washed with ethanol, the asexual conidia were removed, and black, spherical sclerotia were visible on the wild‐type plates. However, there were no sclerotia produced by any of the mutant colonies, including the *hamE* mutant. Complementation of each gene resulted in the restoration of the wild‐type phenotype, as each of these strains were capable of undergoing normal asexual conidiation and sexual sclerotia formation. The average percentages of sclerotia production for each complementation strain in comparison to the wild type average were between 71.24%–132.42% (Figure 3b).

qPCR analysis was performed to assess the relative expression levels of various sexual development genes in an *mkkB* mutant, in comparison to a wild‐type strain (Figure 3c). The *veA* and *nsdD* genes were significantly decreased in the mutant strain (0.5 and 0.62 fold of controlm respectively), while expression of the *velB* and *laeA* genes did not exhibit any significant differences.

Overall, these data suggest that each member of the pheromone module pathway is essential for the regulation of sexual development and sclerotia formation, similar to how these proteins regulate sexual development and cleistothecia formation in *A. nidulans* (Frawley et al., 2018). While HamE was also shown to be critical for sclerotia production, perhaps, this protein does not directly function within the pheromone module pathway to regulate this process.

### 2.4 The production of various SMs is dependent on the pheromone module proteins

Due to the defects in development observed in the pheromone module mutant strains, we decided to assess whether these proteins contribute to the regulation of secondary metabolism, similar to what is observed in *A. nidulans* (Bayram et al., 2012; Frawley et al., 2018). *A. flavus* is capable of producing a wide range of SMs including the carcinogen aflatoxin B1(Klich, 2007), the antiinsectant/antifeedant leporin B (Cary et al., 2015), the indole‐tetramic acid mycotoxin cyclopiazonic acid (CPA) (Chang, Horn, & Dorner, 2009), the innate immunity modulators aspergillicin A and aspergillicin F (Capon et al., 2003; Greco, Pfannenstiel, Liu, & Keller, 2019; Kikuchi et al., 2015) and the analgesic/anti‐inflammatory agent ditryptophenaline (Barrow & Sedlock, 1994; Saruwatari et al., 2014).

**Figure 4:**
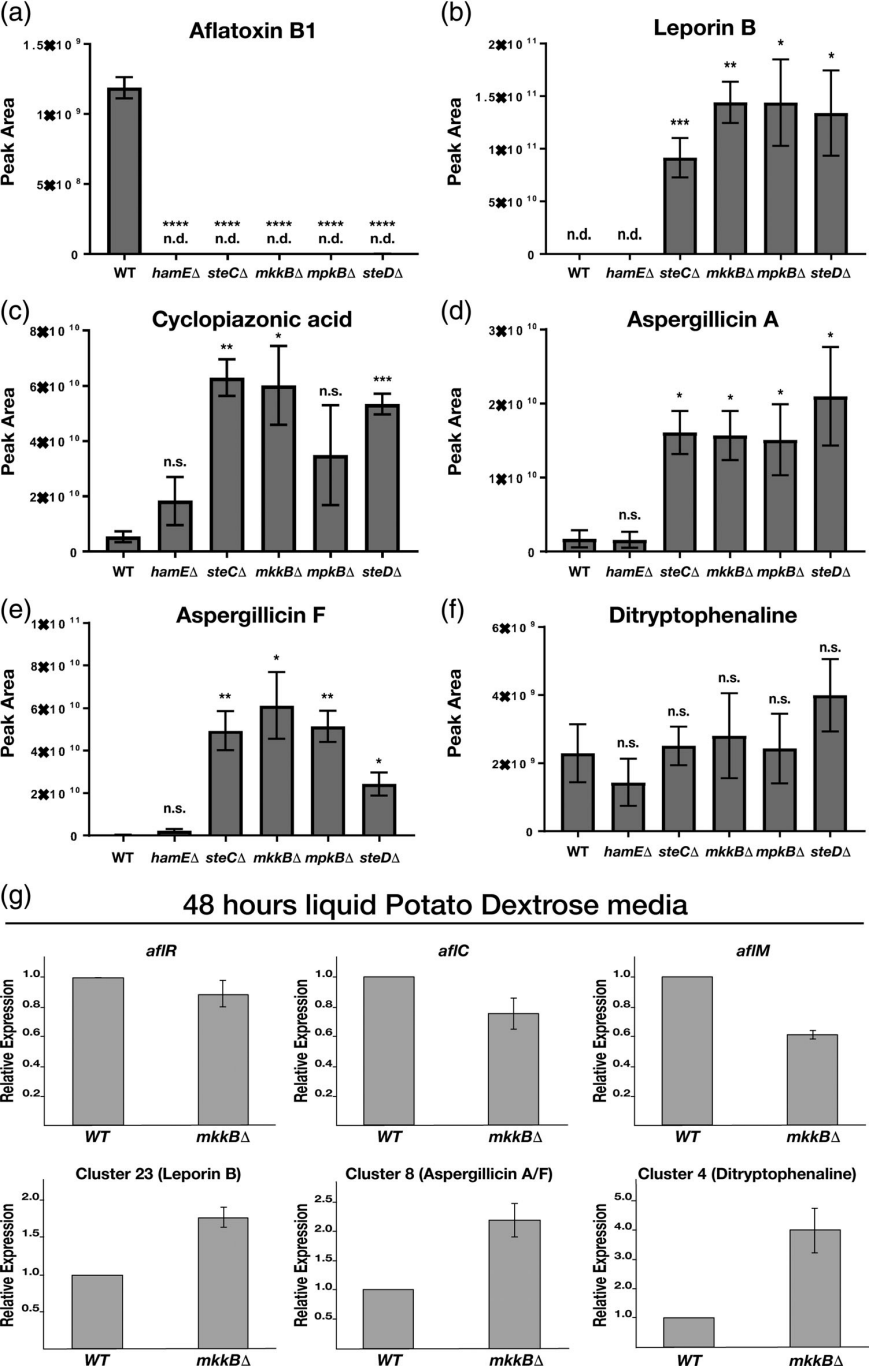
The pheromone module components regulate the production of various secondary metabolites (SMs). (a) Peak area values of aflatoxin B1 detected by UHPLC‐HRMS in the TJES19.1 wild‐type strain and mutant strains. For panels (a–f), strains were point inoculated in triplicate (5 × 10^3^ spores) on PDA plates and incubated in the presence of light for 14 days at 30°C. The bars represent the mean values of three biological replicates per strain ± s.d. *p* values were calculated by performing unpaired Studentʼs *t* tests using the Graphpad Prism Version 6 (*p < .05; **p < .01; ***p < .001; ****p < .0001). n.d. (not detected), n.s. (non‐significant). (b) Peak area values of leporin B. (c) Peak area values of cyclopiazonic acid (CPA). (d) Peak area values of aspergillicin A. (E) Peak area values of aspergillicin F. (f) Peak area values of ditryptophenaline. (g) Relative expression levels of the *aflR*, *aflC* and *aflM* genes belonging to the aflatoxin B1 gene cluster as well as genes corresponding to the leporin B, aspergillicin A/F and ditryptophenaline gene clusters. The TJES19.1 strain and *mkkB*Δ mutant strain were inoculated (2 × 10^6^ spores) in liquid PDB media and left to incubate on a shaker at 30^°C^ for 48 hr. mRNA was isolated from two independent biological replicates per strain. Three technical replicates per biological replicate were used (N = 6). The average expression level values were plotted ± s.d. as a percentage of the wild type average, which was chosen to represent 100% expression (1.0 relative expression). *skpA* was used as a reference gene to obtain relative expression levels of corresponding genes using the 2^ΔΔCt^ method

Ultra‐high‐performance liquid chromatography high resolution mass spectrometry (UHPLC‐HRMS) analysis was performed to test whether the deletion of any of the pheromone module proteins influences the production of the six metabolites mentioned earlier. Interestingly, it was found that the *steC*, *mkkB*, *mpkB* and *steD* mutants exhibit very similar metabolic profiles that differ significantly from the wild type and *hamE*Δ strain (Figure 4a–f, Figure S3 and S4). It was observed that each of the five mutants were incapable of producing aflatoxin B1 (Figure 4a). The production of leporin B (Figure 4b), CPA (Figure 4c), aspergillicin A (Figure 4d) and aspergillicin F (Figure 4e) was increased in the *steC*, *mkkB*, *mpkB* and *steD* mutants, whereas the deletion of *hamE* did not result in any significant differences. With regard to ditryptophenaline production (Figure 4f), there were no significant changes observed in any of the mutant strain samples with respect to the wild‐type strain.

To determine the relative gene expression levels of genes that contribute to the biosynthesis of the SMs tested by UHPLC‐HRMS, qPCR analysis was performed (Figure 4g). The *aflR* (AFLA_139360), *aflC* (AFLA_139410) and *aflM* (AFLA_139300) genes belonging to the aflatoxin B1 biosynthetic gene cluster were tested in the wild type and *mkkB* mutant strain. It was observed that the relative expression levels of both *aflR* and *aflC* exhibited no significant differences in the *mkkB* mutant, while a significant reduction in expression of the *aflM* gene was detected (0.61 fold of control). The backbone genes of leporin B (AFLA_066840‐*lepA*), CPA (AFLA_139490‐*cpaA*), aspergillicin A/aspergillicin F (AFLA_010580‐*agiA*) and ditryptophenaline (AFLA_005440‐*dtpA*) were also tested. It was found that the relative expression levels of *lepA*, *agiA* and *dtpA* were upregulated (1.79, 2.18 and fourfold increase respectively) in the *mkkB* mutant. The *cpaA* gene was not detectable at this time point.

Taken together, these data show that deletion of either *steC*, *mkkB*, *mpkB* or *steD* results in abolishment of aflatoxin B1 production and an increase in leporin B, CPA, aspergillicin A and aspergillicin F production. However, while the deletion of *hamE* results in the complete loss of aflatoxin B1 production, it exhibits no significant differences with respect to production of the five other compounds tested. This could suggest that HamE does not function as a member of the pheromone module in *A. flavus* to regulate SM production, but it may independently regulate aflatoxin production via a separate mechanism.

### 2.5 The pheromone module is assembled in the cytoplasm and MpkB translocates into the nucleus

**Figure 5:**
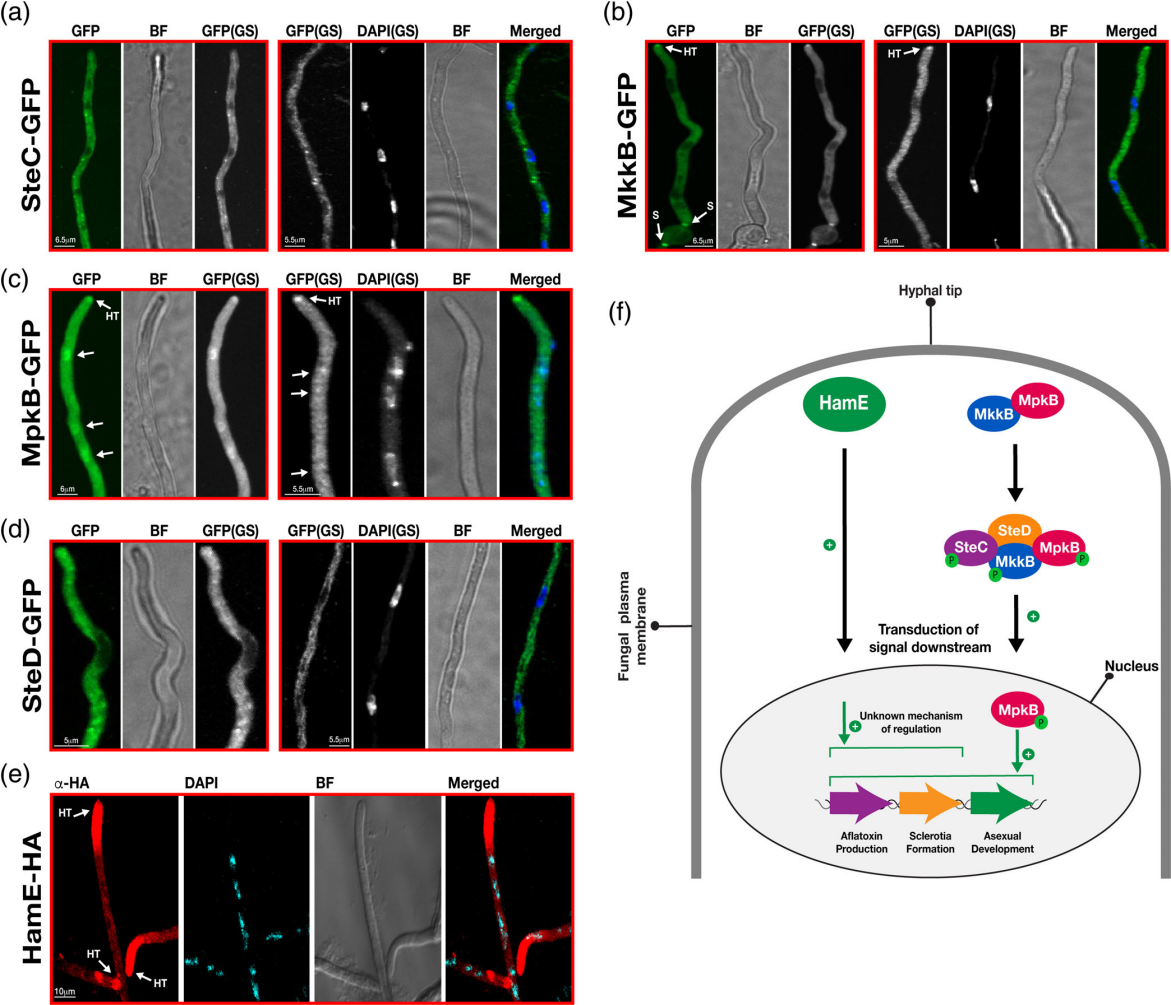
The sub‐cellular localizations of the pheromone module proteins and HamE in vivo. (a) Sub‐cellular localization of SteC‐GFP. All strains (panels a–d) were incubated at 30°C for various durations (less than 24 hr) in 400 μL of liquid GMM, containing appropriate supplements. (BF) refers to brightfield images. (GS) refers to grayscale images. DAPI was used to stain the nuclei. White arrows refer to accumulation of protein in the nuclei. (HT) refers to accumulation of protein at hyphal tips. (S) refers to accumulation of protein at septa. (b) Sub‐cellular localization of MkkB‐GFP. (c) Sub‐cellular localization of MpkB‐GFP. (d) Sub‐cellular localization of SteD‐GFP. (e) Localization of HamE‐HA in vivo. The HamE‐HA strain was inoculated (5 × 10^3^ spores) on sterile coverslips, covered in 450 μL of Sabouraud media, containing supplements. Strains were left to incubate at 30°C for 14–16 hr. Scale bars represent 10 μm. (f) Schematic model of the pheromone module in *Aspergillus flavus*. The MkkB‐MpkB dimer, as well as HamE, localize to the hyphal tips. The MkkB‐MpkB dimer likely interacts with the SteC‐SteD dimer in the cytoplasm to form a tetrameric complex. Both HamE and the tetrameric complex signal downstream to the nucleus independently. Assembly of the complex leads to activation of MpkB, possibly by phosphorylation, which then results in translocation of MpkB into the nucleus. Presumably, MpkB then interacts with transcription factors to positively regulate asexual sporulation, sexual sclerotia formation and aflatoxin B1 production. HamE positively regulates sclerotia formation and aflatoxin B1 production via an unknown mechanism. “P” represents phosphate groups

To determine the sub‐cellular localizations of the pheromone module proteins in vivo, confocal microscopy imaging was performed using the GFP‐tagged proteins. Strains were imaged initially without DAPI staining to observe the localizations of these proteins in living material. Samples were later fixed and stained with DAPI to compare the localizations of these proteins with respect to the nuclei. It was observed that SteC‐GFP exhibited uniform cytoplasmic fluorescence throughout hyphae and is excluded from interphase nuclei. It was also evident that SteC‐GFP localizes to some but not all septa (Figure 5a). MkkB‐GFP displayed a uniform distribution throughout fungal hyphae. It was observed that this fusion protein is excluded from nuclei but is enriched at the septa and hyphal tips (Figure 5b). Imaging of MpkB‐GFP revealed that this fusion protein is localized throughout the fungal hyphae, exhibiting a uniform distribution. MpkB was also observed to be slightly more concentrated in the nuclei and at the hyphal apices (Figure 5c). SteD‐GFP fluorescence was faint, cytoplasmic and non‐uniform. This fusion protein was observed to accumulate at some but not all septa, and it appears that it is excluded from nuclei (Figure 5d). To visualize the sub‐cellular localization of HamE‐HA in vivo, immunostaining was performed. The HamE‐HA fusion protein is dispersed throughout the fungal hyphae and becomes enriched at the hyphal tips and is excluded from the nuclei (Figure 5e), similar to what is observed in *Neurospora crassa* (Dettmann, Heilig, Valerius, Ludwig, & Seiler, 2014; Jonkers et al., 2014).

Taken together, these data show that both MkkB and MpkB accumulate at the hyphal tips, perhaps forming a dimer in response to pheromone signalling between neighbouring hyphae. Both SteC and SteD have been shown to physically interact and were dispersed throughout the hyphae. Thus, it is likely that these two proteins form a dimer in the cytoplasm. Assembly of a cytoplasmic tetrameric complex of SteC, MkkB, MpkB and SteD could allow for kinase phosphorylation and translocation of MpkB into the nucleus. This would result in the activation of various transcription factors to regulate asexual sporulation, sexual sclerotia formation and aflatoxin B1 production. These data also suggest that HamE is enriched at the hyphal tips and may be involved in signalling via an unknown mechanism to the nucleus to regulate both sclerotia formation and aflatoxin B1 production, but not asexual sporulation (Figure 5f).

## 3 DISCUSSION

MAP kinase cascades are highly conserved signalling pathways in eukaryotes that are responsible for the regulation of a wide array of biological processes (Schaeffer & Weber, 1999). In the model filamentous fungus *A. nidulans*, a MAPK cascade known as the pheromone module is assembled in response to pheromone signalling between hyphae. This pathway consist MAP3K SteC, MAP2K MkkB, MAPK MpkB, the adaptor protein SteD and the multi‐domain scaffold protein HamE, which together form a pentameric complex. (Bayram et al., 2012; Frawley et al., 2018). Characterization of this complex led to the discovery that the pheromone module is utilized to regulate both asexual sporulation, sexual cleistothecia formation and the production of various SMs such as the carcinogen sterigmatocystin, the antibiotic penicillin and the anti‐tumour compound terrequinone A.

Genes encoding the pheromone module proteins have been found to be highly conserved within filamentous fungal genomes (Rispail et al., 2009). This led to the hypothesis that other filamentous fungi such as *A. flavus* may utilize the pheromone module to regulate their development and secondary metabolism. In this study, we identified homologs of each of the five *A. nidulans* pheromone module proteins in *A. flavus* (Figure 1a and b). Via a genetic and proteomic approach, we have shown that the three kinases and the adaptor protein SteD physically interact to form a tetrameric complex (Figure 1c,d, Tables S4–S7), which is likely made up of two sub‐complexes. MkkB and MpkB possibly form a dimer that becomes enriched at the hyphal tips, and these proteins may then interact with the SteC‐SteD dimer to form a cytoplasmic tetrameric complex (Figure 5a–d), perhaps in response to pheromone signalling between neighbouring hyphae. Assembly of the complex possibly results in kinase phosphorylation and translocation of MpkB into the nucleus (Figure 5c), similar to what is observed in *A. nidulans* (Bayram et al., 2012). Presumably, MpkB would then interact with various transcription factors to regulate a myriad of biological processes, as is observed in other fungal species (Bayram et al., 2012; Dettmann et al., 2014; Li et al., 2005; van Drogen et al., 2001). Interestingly, HamE was not found to interact with any members of the pathway (Table S8) and was shown to accumulate at the hyphal tips (Figure 5e). However, the distribution of HamE at the hyphal tips was different from MkkB and MpkB. This suggests that HamE may be involved in responding to signalling between hyphae but may not act as a scaffold in the *A. flavus* pheromone module. Perhaps the adaptor SteD is exerting a scaffolding role in this MAPK pathway, although further testing would be required to confirm this claim.

This study has shown that the proteins of the pheromone module contribute to the regulation of both asexual sporulation and sexual sclerotia formation (Figures 2 and 3). Similarly to what is observed in *A. nidulans*, the presence and absence of light induces asexual and sexual development in *A. flavus*, respectively (Adams, Wieser, & Yu, 1998; Amaike & Keller, 2011; Purschwitz et al., 2008). In the presence of light, each of the *A. flavus* kinase mutants, as well as the *steD* mutant, exhibited dramatic reductions in asexual sporulation (Figure 2a,c), which complement the findings in *A. nidulans* (Bayram et al., 2012; Frawley et al., 2018). Additionally, the relative expression levels of the asexual development genes *flbC* and *abaA* were significantly downregulated in an *mkkB* mutant (Figure 2d). Interestingly, the deletion of *hamE* did not result in any significant defects in sporulation with regards to the wild type. This largely supports a previous study of the role of *ham* genes on fusion processes in *A. flavus* where loss of *hamE* had relatively small contributions to sporulation but a large impact on sclerotia, as found here (Zhao et al., 2017).

In the absence of light, it was observed that all mutants, including the *hamE* deletion, resulted in the complete abolishment of sclerotia formation (Figure 3a,b). This is similar to what is observed in both *A. nidulans* (Bayram et al., 2012; Frawley et al., 2018) and *N. crassa* (Dettmann et al., 2014). In these species, the deletion of various pheromone module proteins and *hamE/ham5* results in reduced levels of cleistothecia and protoperithecia, which are both sexual reproductive structures. Furthermore, in *A. flavus*, it was shown that the relative expression levels of the sexual development genes *veA* and *nsdD* were significantly downregulated in an *mkkB* mutant (Figure 3c). Together, these phenotypical data provide further evidence for the existence of a tetrameric complex consisting of the three kinases and the adaptor SteD, as each of these mutants displayed highly similar asexual and sexual phenotypes. However, it is likely that HamE is not required for the regulation of asexual sporulation and may function independently of the pheromone module to regulate sclerotia production.

UHPLC‐HRMS and qPCR analysis revealed that the pheromone module proteins are required for the positive and negative regulation of a wide variety of SMs. *A. flavus* is capable of producing a myriad of both beneficial and detrimental SMs, such as the carcinogenic aflatoxins (Klich, 2007; J. Yu et al., 2005), which can lead to crop contamination and development of hepatocellular carcinomas or aflatoxicoses (Bhatnagar‐Mathur et al., 2015; Kew, 2013; Lewis et al., 2005; Liu & Wu, 2010). The deletion of *steC*, *mkkB*, *mpkB* or *steD* results in highly similar metabolic profiles which differ significantly with respect to the wild‐type strain, whereas the deletion of *hamE* mostly resembles the wild type (Figure 4a–f, Figure S4). Regarding aflatoxin B1 production, it was found that all five mutants exhibit undetectable levels of this compound (Figure 4a). However, the levels of leporin B, CPA, aspergillicin A and aspergillicin F are all significantly increased in the *steC*, *mkkB*, *mpkB* and *steD* mutants, but not the *hamE* mutant (Figure 4b–e). qPCR analysis also shows that the deletion of *mkkB* results in the slight downregulation of genes belonging to the aflatoxin gene cluster as well as the upregulation of genes that correspond to the production of leporin B, aspergillicin A and aspergillicin F (Figure 4g). Together these data suggest that SteC, MkkB, MpkB and SteD function in a similar manner to regulate the production of various SMs. This data also propose that HamE is essential for aflatoxin production but may exert its regulatory role independently of the pheromone module. A previous study showed *hamE* was regulated by NosA, a transcription factor involved in sexual development in *A. nidulans* (Vienken & Fischer, 2006) via LaeA activation (Zhao et al., 2017).

In conclusion, this study has identified homologs of the pheromone module proteins in the filamentous fungus *A. flavus*. Data from this study provide evidence to support the assembly of a cytoplasmic tetrameric MAPK signalling pathway, perhaps in response to chemotropic interactions between hyphae. This protein complex consists three kinases SteC, MkkB and MpkB, as well as the adaptor protein SteD (Figure 5f). Assembly of the complex may result in kinase phosphorylation and MpkB activation, allowing for its translocation into the nucleus where it interacts with various transcription factors to positively regulate asexual sporulation, sexual sclerotia formation and aflatoxin B1 production. It can be speculated that in the absence of any of the three kinases or SteD, the tetrameric pheromone module may either not assemble or kinase phosphorylation could be inhibited, resulting in the negative regulation of various transcription factors and biological processes.

These data also suggest that this complex does not require HamE to regulate MAPK signalling, unlike what is observed in other fungal species like *A. nidulans* (Frawley et al., 2018) and *N. crassa* (Dettmann et al., 2014; Jonkers et al., 2014), where HamE acts as a scaffold, regulating kinase phosphorylation and signal transduction to the nucleus. However, HamE was observed to accumulate at the hyphal tips and is critical for both sclerotia formation and aflatoxin B1 production. Perhaps HamE is also required for the response to chemotropic signals, in a manner that is unique to *A. flavus*. It is possible that HamE may be required as a scaffold during the later stages of sclerotia formation. However, the mechanism of HamE‐dependent signalling to the nucleus, as well as the direct functions of HamE, is not fully understood.

By characterizing the molecular roles of the pheromone module in *A. flavus*, this may provide insight on how filamentous fungi regulate their development and secondary metabolism. This, in turn, may allow for strategies to be established that could result in the prevention of crop spoilage due to mycotoxin contamination and infections caused by *Aspergillus* species.

## 4 EXPERIMENTAL PROCEDURES

### 4.1 Strains and growth media

All fungal strains that were used in this study are listed in Table S1. The *A. flavus* TJES19.1 strain served as a wild‐type host for all epitope taggings and deletions. Various plasmids and primers used for the epitope tagging and deletion experiments are listed in Tables S2 and S3, respectively. Plasmids were cloned into Stellar (Clontech) competent *Escherichia coli* cells. Competent cells were cultured in LB media (supplemented with 100 μg/mL ampicillin) and SOC media. For the asexual and sexual growth of fungal strains, the following agar media were used: Glucose Minimal Media (GMM), Potato Dextrose Agar (PDA) and Wickerham (WHM). For vegetative growth, liquid complete media and Sabouraud media were used. Details of the ingredients of each media are found in the supplementary information provided.

### 4.2 Phenotypic assays

Strains were point inoculated (5 × 10^3^ spores) in triplicate on PDA or WHM agar plates containing appropriate supplements. Strains inoculated on PDA plates were incubated in the presence of light for 5 days to induce asexual sporulation, while strains inoculated on WHM agar plates were incubated in the absence of light for 2 weeks to induce sclerotia production. All incubations were performed at 30°C. An Olympus szx16 microscope with Olympus sc30 camera was used to capture stereomicroscopic images. The Cell Sens Standard software (Olympus) was used to take and process digital pictures. Quantifications of colony diameter, asexual conidiation and sclerotia production were performed using three independent biological replicates. Bar charts represent the mean values ± s.d. *p* values were calculated by performing unpaired Studentʼs *t* tests (*p < .05; **p < .01) using the Graphpad Prism Version 6.

### 4.3 GFP/HA‐trap and sample preparation for LC–MS protein identification

The isolation and preparation of both GFP and HA fusion proteins for mass spectrometry analysis were performed as explained in detail (Bayram et al., 2008). Detailed descriptions of methods used are given in the provided supplementary information.

### 4.4 mRNA extractions and quantitative PCR analysis

Per replicate, 100–150 mg of mycelia was collected, and mRNA was isolated according to the “RNeasy Plant Mini Kit” protocol (Qiagen). mRNA was quantified with the “Qubit RNA BR Assay Kit” Protocol (Thermo Fisher). 1 μg of mRNA per replicate was used to synthesize cDNA using the “Transcriptor First Strand cDNA Synthesis Kit” (Roche). To prepare qPCR reaction mixtures, the LightCycler 480 SYBR Green I Master mix was used. A LightCycler 480 qPCR machine (Roche) was used to determine gene expression levels, using the *skpA* control gene as a reference. Bar charts represent the mean data of two combined biological replicates and six combined technical replicates per strain, ± s.d. Additional details of the culture conditions and qPCR cycle parameters are provided in the supplementary information.

### 4.5 Sample preparation and UHPLC‐HRMS of *A. flavus* metabolites

Strains were spot inoculated (5 × 10^3^ spores) in triplicate on PDA plates containing supplements and were left to incubate in the presence of light at 30°C for 2 weeks. Half of each agar plate was cut into small pieces using a scalpel and placed into 40 mL glass vials. 10 mL ethyl acetate was added to each vial, and samples were sonicated for 90 min. 10 mL water was then added to each sample, and the vials were shaken for 5 s. Samples were left at room temperature for 10 min to allow for separation of the two layers. 10 mL of the ethyl acetate layer was removed, using a glass pipette and was transferred to pre‐weighed 12 mL glass vials. Samples were left to dry in a fume hood, and the crude extracts were weighed.

The crude extracts were re‐suspended in acetonitrile (10 mg/mL) and filtered through an Acrodisc syringe filter with a nylon membrane (Pall Corporation) (0.45 μm pore size). Ultra‐high‐performance liquid chromatography coupled with high resolution mass spectrometry (UHPLC‐HRMS) was then performed on a Thermo Scientific‐Vanquish UHPLC system connected to a Thermo Scientific Q‐Exactive Orbitrap mass spectrometer in ES^+^ mode between 200 m/z and 1,000 m/z to identify metabolites. A Zorbax Eclipse XDB‐C18 column (2.1 × 150 mm, 1.8 μm particle size) was used with a flow rate of 0.2 mL/min for all samples. LCMS grade water with 0.5% formic acid (solvent A) and LCMS grade acetonitrile with 0.5% formic acid (solvent B) were used with the following gradient 0 min, 20% Solvent B; 2 min, 20% Solvent B; 15 min, 95% Solvent B; 20 min, 95% Solvent B; 20 min, 20% Solvent B; 25 min, Solvent B. Nitrogen was used as the sheath gas. Data acquisition and procession for the UHPLC–MS were controlled by Thermo Scientific Xcalibur software. Files were converted to the .mzXML format using MassMatrix MS Data File Conversion, and analysed in MAVEN and XCMS (Clasquin, Melamud, & Rabinowitz, 2012). The peak area (from three biological replicates per strain) is presented as a bar chart with the bars representing the mean ± s.d. *p* values were calculated by performing unpaired Studentʼs *t* tests using the Graphpad Prism Version 6.

### 4.6 Confocal microscopy

For confocal microscopy imaging, conidia were cultured in eight‐chambered cover glasses (Lab‐Tek; Thermo Fisher Scientific). Strains were incubated at 30°C for various durations in 400 μL of liquid GMM, containing appropriate supplements. Additional details for DAPI staining and confocal imaging are provided in the supplementary information.

### 4.7 Immunostaining

Coverslips were soaked in 100% ethanol for 10 s and flame sterilized. Sterile coverslips were then added to a 6‐well macrotitre tray and 450 μL of Sabouraud media (containing supplements) was added to each coverslip. 5 × 10^3^ spores of each strain were used to inoculate the media and strains were left to incubate at 30°C for 14–16 hr. Additional details for the immunostaining protocol and confocal imaging are provided in the supplementary information.

## Supporting information


**Appendix S1**. Supporting information.
