# Supplementary material for: The tetrameric pheromone module SteC‐MkkB‐MpkB‐SteD regulates asexual sporulation, sclerotia formation and aflatoxin production in Aspergillus flavus
Source: Cell Microbiol. 2020 Mar 3;22(6):e13192. doi: 10.1111/cmi.13192 (PMC7202998; doi:10.1111/cmi.13192)
Supplement: Supplementary file 1 — Appendix S1. Supporting information. [file CMI-22-e13192-s001.docx]

**Supplementary Information**

**The tetrameric pheromone module SteC-MkkB-MpkB-SteD regulates asexual sporulation, sclerotia formation and aflatoxin production in *Aspergillus flavus***

Dean Frawley*, Claudio Greco^†^, Berl Oakley^‡^, Mohamed M. Alhussain^§^,

Alastair B. Fleming^§^, Nancy P. Keller^†,^**, Özgür Bayram*^1^

* Biology Department, Maynooth University, Maynooth, Co. Kildare, W23 F2H6, Ireland.

^†^ Department of Medical Microbiology and Immunology, University of Wisconsin-Madison, Madison, Wisconsin, United States.

^‡^ Department of Molecular Biosciences, University of Kansas, 1200 Sunnyside Avenue, Lawrence, Kansas, United States of America.

^§^ Department of Microbiology, School of Genetics and Microbiology, Moyne Institute of Preventive Medicine, Trinity College Dublin, Dublin, Ireland.

** Department of Bacteriology, University of Wisconsin-Madison, Madison, Wisconsin, United States.

Running title: The pheromone module in *Aspergillus flavus*

^1^ Correspondence: Dr. Ozgur Bayram.

Fungal Genetics and Secondary Metabolism Laboratory, Biology Department, Maynooth University, Maynooth, Co. Kildare, W23 F2H6, Ireland.

Email: [ozgur.bayram@mu.ie](mailto:ozgur.bayram@mu.ie). Contact Number: (01) 708 6879

**Supplementary methods**

**Fungal strains, growth media and culture conditions**

All fungal strains that were used in this study are listed in Table S1. The *Aspergillus flavus* TJES19.1 strain served as a wild type host for all epitope taggings and deletions. All plasmids used for the deletion, complementation and epitope tagging experiments are listed in Table S2. All plasmids were cloned into Stellar (Clontech) competent *Escherichia coli* cells. Competent cells were cultured in LB media (1% tryptone, 0.5% yeast extract, 1% NaCl), supplemented with 100 μg/mL ampicillin and SOC media (2% tryptone, 0.5% yeast extract, 10mM NaCl, 2.5mM KCl, 10mM MgCl_2_, 10mM MgSO_4_, 20mM glucose).

For the growth of fungal strains on agar plates, the following media were used: (i) Glucose Minimal Media (GMM) (6 g/L NaNO_3_, 0.52 g/L KCl, 1.52 g/L KH_2_PO_4_, 10 g/L Glucose, 0.24 g/L MgS0_4_, 0.1% trace element solution), (ii) Potato Dextrose Agar (PDA) (P6685 Sigma, 24 g/L), (iii) Wickerham (WHM) (2 g/L yeast extract, 3 g/L peptone, 5 g/L corn steep solids, 2 g/L dextrose, 30 g/L sucrose, 2 g/L NaNO_3_, 0.76 g/L K_2_HPO_4_, 0.24 g/L MgS0_4_, 0.2 g/L KCl, 0.1 g/L FeSO_4_∙7H_2_O). Liquid media used were complete media (GMM media ingredients with the addition of 1 g/L tryptone, 2 g/L peptone and 1 g/L yeast extract) and Sabouraud media (CMO147 Oxoid, 30 g/L). All appropriate supplements were added to media prior to inoculation with fungal spores. These supplements included uracil (1 g/L), uridine (0.25 g/L) and phleomycin (100 µg/mL).

All fungal strains inoculated on agar plates and liquid media were cultured at 30 ^°^C. Agar plates were either incubated in the presence or absence of light for various durations depending on the experiment. Strains inoculated in liquid media were left to incubate on a shaker at 180 RPM for various durations.

**Construction of plasmids**

Details of all plasmids used in this study are provided in Table S2 and all oligonucleotide sequences are provided in Table S3. To design all plasmid maps *in silico*, the Lasergene Seqbuilder software was used. For all cloning experiments the following plasmids were used: (i) pUC19 (Fermentas) digested with a *Sma*I restriction enzyme (Thermo Scientific), (ii) pAN8-1 digested with a *Stu*I restriction enzyme (Thermo Scientific) and (iii) pOSB113 digested with a *Swa*I restriction enzyme (Thermo Scientific). To construct all fragments required for gene knock-outs, 1-2 kb 5’ UTR and 3’ UTR flanking regions of the gene of interest were PCR-amplified from genomic DNA with respective oligonucleotides. These UTR fragments were fused by fusion PCR to a selection marker (*pyrG, phleO*) that was PCR-replicated from a plasmid containing the marker of interest. These three fragments were then cloned into the *Sma*I site of pUC19 by In-Fusion HD Cloning enzyme (Clontech, 121416).

To create the *sgfp* and *3xha* fusion constructs, 1.5-2 kb of the gene ORF (with stop codon removed) and 1-1.5 kb of the 3’ UTR sequences were PCR-amplified from genomic DNA and fused to either *sgfp* or *3xha* epitope tags connected to selection markers (*pyrG, phleO*). All epitope tags were fused to the C-terminal ends of the gene ORFs. These three fragments were then cloned into the *Sma*I site of pUC19 and transformed into competent bacterial cells.

To create the complementation constructs, primers were created to replicate DNA fragments containing an entire gene ORF and 1.5-2 kb of both the 5’ and 3’ UTRs from a genomic DNA template. These genomic fragments were then cloned into plasmids that contain various selection markers. The *A. flavus hamE* complementation construct was cloned into the *Stu*I site of the pAN8-1 plasmid which contains a phleomycin resistance cassette (*phleO*), while the *A. flavus steC, mkkB, mpkB* and *steD* complementation constructs were cloned into the *Swa*I site of the pOSB113 plasmid, which contains a *pyrG* marker. All ligated complementation plasmids were then transformed into competent bacterial cells. 10-15 μg of each plasmid was transformed ectopically into fungal deletion recipient strains.

**Bacterial and fungal cell transformation**

150 μL of competent Stellar *E. coli* cells were added to ligated circular plasmid constructs. These samples were then left on ice to incubate for 30 minutes. Samples were then heat shocked at 42 ^°^C for 1 minute and immediately put back on ice. To each sample, 800 μL SOC media was added and samples were left to incubate on a shaker for 1 hour at 37 ^°^C. Samples were then centrifuged at 13,000 RPM for 1 minute. The supernatant was removed and bacterial cells were spread on LB agar plates containing ampicillin. Plates were left to incubate at 37 ^°^C for 14-16 hours.

For the transformation of fungal cells, strains were cultured at 30^o^C for 24 hours in liquid complete media or Sabauraud medium. The mycelia were filtered through sterile miracloth and washed three times with citrate buffer (150 mM KCl, 580 mM NaCl, 50 mM sodium citrate, pH5.5). Mycelia were transferred to sterile flasks and incubated in enzyme solution (20 mL citrate buffer containing 500 mg glucanase (Novozymes) and 240 mg lysozyme (Serva) for 100 minutes at 30 ^°^C. Resulting protoplasts were then filtered through sterile miracloth into a 50 mL Falcon tube. Pre-chilled STC1700 (1.2 M sorbitol, 10 mM Tris pH5.5, 50 mM CaCl_2_, 35 mM NaCl) was added to each Falcon tube to make the volume up to 50 mL. Samples were then inverted repeatedly and left to incubate on ice for 10 minutes. Samples were centrifuged at 4 ^°^C for 15 minutes at 2,600 RPM. The supernatant was removed and the protoplast pellet was resuspended in 1 mL STC1700 buffer. The volume was again made up to 50 mL with STC1700 buffer and samples were centrifuged using the same settings. The supernatant was then removed and again the protoplast pellet was resuspended in 1 mL STC1700. Protoplasts were separated into 2x200 μL aliquots, with 1 μg of linear DNA or 10-15 μg of circular plasmid DNA being added to one aliquot and no DNA being added to the other, to act as a negative control. Samples were incubated on ice for 20-30 minutes. PEG4000 (60% PEG4000, 10mM Tris pH7.5, 50mM CaCl_2_) was added to each sample three times (2 x 250 μL and 1 x 850 μL aliquots). Following each addition, samples were inverted 20 times. Samples were left to incubate in a rack on top of ice for 40 minutes. STC1700 was added to each falcon tube to make the volume up to 15 mL and samples were inverted. Samples were centrifuged at 4 ^°^C for 15 minutes at 2,600 RPM. The supernatant was then discarded, the pellet was resuspended and protoplasts were inoculated on GMM agar plates containing 1.2 M sorbitol and incubated at 30 ^°^C.

**Hybridization techniques**

To design all 5’ and 3’ UTR probes for hybridisation, the Lasergene SeqBuilder software was used. This software also allowed for the selection of appropriate restriction enzymes for digesting genomic DNA. Probes were synthesised and labelled with Digoxigenin-11-UTP (PCR DIG Probe Synthesis Kit: Roche), using either 5’ or 3’ UTRs as templates and the respective oligonucleotides. To isolate fungal genomic DNA, the ‘Zymo Research Fungal/Bacterial Miniprep Kit’ was used. 700 ng of DNA was digested overnight with various restriction enzymes and the digested DNA samples were separated on a 0.7% agarose gel at 100 Volts for 90 minutes. The gel was then washed 3 times on a shaker at room temperature in the following solutions: (i) 0.25 M HCl for 10 minutes, (ii) 0.5 M NaOH/1.5 M NaCl for 25 minutes and (iii) 1.5 M NaCl/0.5 M Tris for 30 minutes. The DNA fragments were then transferred and UV cross-linked (UV Stratalinker 1800) to a nylon membrane (Amersham Hybond^TM^-N^+^, GE Healthcare). The membrane was washed twice with 2x SSC (Saline Sodium Citrate) buffer and dried for 5 minutes at 70 ^°^C. The membrane was incubated in a rotating tube at 42 ^°^C in pre-hybridisation buffer for 1 hour on a rotator. The probe was then added and left to incubate overnight.

The next day, the membrane was washed with 2x SSC/0.1% SDS solution for 5 minutes, followed by 2 washes with pre-heated 0.1x SSC/0.1% SDS for 20 minutes. The membrane was then washed with 20 mL DIG buffer 1, followed by incubation in 15 mL DIG buffer 2 for 30 minutes. Alkaline phosphatase conjugated anti-DIG fab fragment (Roche 11093274910) was then added to the DIG buffer 2 (1:10,000 dilution) and left to incubate for 1 hour. 2 washes with 20 mL wash buffer were performed for 15 minutes, followed by an incubation in 10 mL DIG buffer 3 for 5 minutes. For chemiluminescent detection, CDP Star substrate (Roche) was added to the membrane and the membrane was exposed using the G:BOX Chemi XRQ (Syngene).

**Protein extraction and immunoprecipitation of tagged proteins**

All crude protein extracts were isolated from vegetative cultures that were incubated in liquid complete media on a shaker overnight. Liquid nitrogen was used to break the mycelia. To lyse cells and isolate proteins, 300-400 μL of protein extraction buffer was added to each sample of mycelia. The protein extraction buffer recipe is as follows: (300 mM NaCl, 50 mM Tris-HCl pH 7.5, 10% glycerol, 1 mM EDTA, 0.1% NP-40) that has been supplemented with 1 mM DTT, 1X complete EDTA-free protease inhibitors (Roche), 1 mM benzamidine, 0.5 mM PMSF and 1X phosphatase inhibitors (1 mM NaF, 0.5 mM sodium orthovanadate, 8 mM β-glycerol phosphate) immediately prior to use. Resuspended mycelial samples were centrifuged at 13,000 RPM for 15 minutes at 4 ^°^C. 1 mL of the protein supernatant was then transferred to a new 1.5 mL microcentrifuge tube.

For the immunoprecipitation of GFP and HA fusion proteins, 10 μL GFP-Trap sepharose (Chromotek) and 10 μL anti-HA magnetic beads (Pierce) were washed twice with 190 μL protein extraction buffer, containing supplements (as described above). The anti-GFP/HA beads were then resuspended in 50 μL protein extraction buffer and added to 1 mL crude protein extract. This mixture was left to incubate on a rotator at 4 ^°^C for 3 hours. Samples were placed in a magnetic rack to collect the beads and the supernatant was discarded. Beads were washed twice with 1 mL protein extraction buffer (without supplements) and were then washed for a third time with the same buffer containing 1 mM DTT. All liquid was removed and the beads were stored at -80 ^°^C until further use.

**Preparation of tagged protein samples for LC-MS/MS identification**

GFP and HA-tagged proteins that had been isolated were resuspended in 50 mM ammonium bicarbonate. 1 μL of 0.5M DTT was then added to each and samples were incubated for 20 minutes at 56 ^°^C. 2.7 μL of iodoacetamide (0.55 M) was then added and samples were incubated for 15 minutes in the dark. 1 μL of 1% (w/v) ProteaseMAX (Promega) was added, followed by addition of 1 μL trypsin (1 μg/μL) (Promega). Samples were incubated at 37 ^°^C overnight. The next day, 1 μL of Trifluoroacetic acid (TFA) was added to each and samples were vortexed briefly and left to incubate for 5 minutes at room temperature. To isolate and remove the beads, samples were placed on a magnetic rack and the supernatant was transferred to new tubes. The supernatants were centrifuged for 10 minutes at 13,000 RCF and dried in a speed-vacuum for 2-3 hours. Samples were stored at -20^o^C until further use.

Peptide samples were resuspended in 20 μL resuspension buffer (0.5% TFA) and sonicated for 3 minutes, followed by a brief centrifugation. ZipTip C_18_ pipette tips (Millipore) were used to purify peptide samples prior to mass spectrometric analysis. To equilibrate the ZipTips, a wetting solution (0.1%, 80% acetonitrile) was aspirated 5 times, followed by aspiration of an equilibration buffer (0.1% TFA) 5 times. ZipTips were then used to pipette the peptide samples up and down 15-20 times. Then, the equilibration buffer was aspirated again 5 times, followed by elution of the peptides via aspiration of an elution buffer (0.1% TFA, 60% acetonitrile) 5 times into a new eppendorf tube. This solution was dried in a speed-vacuum for 2 hours and peptide samples were stored at -20 ^°^C.

Immediately prior to loading, peptide samples were resuspended in 15 μL Q-Exactive loading buffer (2% acetonitrile, 0.5% TFA) and 10 μL was added to mass spectrometry vials (VWR). Samples were loaded on a high resolution quantitative LC-MS mass spectrometer (Thermo Fisher Q-Exactive). LC-MS identifications of peptides were performed using the Proteome Discoverer Daemon 1.4 software (Thermo Fisher) and organism-specific taxon-defined protein databases. Unique peptides were determined by isolating only those that do not appear in any of the wild type controls.

**Immunoblotting**

For all immunoblots, protean membranes (0.45 μm pore size, GE Healthcare) were incubated in blocking solution [5% (w/v) non-fat dry milk solution in 1X TBS with 0.1% Tween 20] for 1 hour at room temperature with gentle shaking. For the detection of GFP-tagged proteins, mouse α-GFP antibody (SC-9996, SantaCruz) was used at 1:1,000 dilution in blocking solution for 2 hours at room temperature. Secondary goat α-mouse (170-6516, Biorad) was used at 1:2,000 dilution in blocking solution for 1 hour at room temperature. After each antibody incubation, membranes were washed three times with 1X TBST (0.1% Tween 20) for 5 minutes. For visualisation of all membranes, Luminata Crescendo Western HRP Substrate (Millipore) was added and membranes were exposed using the G:BOX Chemi XRQ (Syngene).

**mRNA extractions and quantitative PCR analysis**

*A. flavus* strains were inoculated either in liquid complete media or liquid PDA media at a concentration of 2x10^6^ spores/ml and were incubated on a shaker at 30 ^°^C. Liquid complete media cultures were left to incubate for 24 hours while liquid PDA cultures were left to incubate for either 48 hours or 72 hours. After 24 hours of incubation in complete media, the mycelia were filtered and transferred onto PDA plates. These plates were left to incubate in the presence of light for 24 hours.

To isolate mRNA, after each incubation the mycelia were filtered through miracloth and washed with DEPC buffer (0.1% DEPC in 1X PBS) three times. 100 mg of mycelia were collected in RNase-free eppendorf tubes and mRNA was isolated according to the ‘RNeasy Plant Mini Kit’ protocol (Qiagen). mRNA was quantified according to the ‘Qubit RNA BR Assay Kit’ Protocol (Thermo Fisher). To synthesise cDNA, 1 μg of mRNA was used for each strain and the ‘Transcriptor First Strand cDNA Synthesis Kit’ (Roche) was used. The final 20 μL cDNA solutions were made up to either 100 μL or 200 μL with PCR-grade water and stored at -20 ^°^C until further use.

For qPCR, cDNA of each duplicate strain was inoculated in triplicate in 96-well plates (Life Science Products). Plates were loaded in a LightCycler 480 qPCR machine (Roche) and the cycle parameters were as follows: Pre-incubation (95 ^°^C, 10 minutes), Amplification [40 cycles] (95 ^°^C-10 seconds, 60 ^°^C-20 seconds, 72 ^°^C-10 seconds), Melting curve (65 ^°^C to 97 ^°^C with continuous fluorescence readings). Advanced relative quantification was used to determine the levels of gene expression in each strain, using a *skpA* control gene as a reference for all strains. Relative expression analysis was performed by using the LightCycler 480 software. Bar charts represent the mean data of two combined biological replicates and 6 combined technical replicates per strain, ± s.d.

**Confocal Microscopy**

For confocal microscopy imaging, conidia were cultured in eight-chambered cover glasses (Lab-Tek; Thermo Fisher Scientific). Strains were incubated at 30 ^°^C for various durations in 400 μL of liquid GMM, containing appropriate supplements.

For 4′,6-diamidino-2-phenylindole (DAPI) staining experiments, germlings were initially fixed in the wells of the chambered cover glasses. To ensure fixation, the liquid medium was removed and 400 μL of fixative solution was added [8 % formaldehyde in 50 mM piperazine-N,N’-bis(2- ethanesulfonic acid) (PIPES), pH 6.7; 25 mM EGTA, pH 7.0; 5 mM MgSO4; and 5 % DMSO, pre-warmed to the culture temperature]. Samples were left to incubate for 30 min at 30 ^°^C. Following this incubation, the fixative solution was removed and replaced with 400 μL of 0.015 μg/mL DAPI dissolved in H_2_O.

To capture images, an UltraView VoX spinning disk confocal system (PerkinElmer) mounted on an Olympus IX71 inverted microscope was utilized. This confocal system is equipped with a piezoelectric stage which is software-controlled to enable rapid Z-axis movement. To collect images, a 60X/1.42 numerical aperture Olympus Plan Apo objective and an ORCA ERAG camera (Hamamatsu Photonics) were used. Solid state 405-nm and 488-nm lasers were used for excitation of DAPI and GFP respectively. For live imaging, the specimen temperature was maintained at 30 ^°^C using a temperature-controlled chamber. For DAPI experiments, fluorochrome-specific emission filters were used to prevent emission bleed through between fluorochromes. The system was controlled by Volocity software (PerkinElmer) running on a Power Mac computer (Apple). A stage micrometer was used to calibrate magnifications. After the adjustment of both minimum and maximum intensity levels (black and white levels) for each channel, the images were exported directly from Volocity.

**Immunostaining**

Coverslips were soaked in 100% ethanol for 10 seconds and flame sterilised. Sterile coverslips were then added to a 6-well macrotitre tray and 450 μL of sabouraud media (containing supplements) was added to each coverslip. 5x10^3^ spores of each strain were used to inoculate the media and strains were left to incubate at 30 ^°^C for 14-16 hours.

The next day, fixation solution was freshly prepared by adding 0.6 g paraformaldehyde to 15 mL PME buffer (50 mM PIPES, 25 mM EGTA, 5 mM MgSO_4_, pH adjusted to 6.7 with NaOH). The solution was left to incubate at 68 ^°^C for 45 minutes until paraformaldehyde had fully dissolved and then solution was left to cool to room temperature before use. All media was removed from wells by using a vacuum pump and 2 mL of fixation solution was added to each coverslip. Samples were left to incubate at room temperature for 30 minutes. All fixation solution was removed using a vacuum pump and coverslips were washed three times for 5 minutes with 2 mL PME buffer, with the liquid being removed after each wash. Lysing enzyme solution was prepared fresh by adding 400 mg of lysing enzymes from *Trichoderma harzianum* (Sigma-L1412) to 20 mL PME buffer. 10 mL of this solution was added to 10 mL egg white and 2 mL of this solution was added to each coverslip. Samples were left to incubate at 25 ^°^C for 50 minutes while shaking slowly (50 RPM). Lysing enzyme solution was then removed and samples were washed three times for 10 minutes with 2 mL PME buffer. 1.5 mL extraction buffer (100 mM PIPES, 25 mM EGTA, 0.1% NP-40) was added to each coverslip and samples were left to incubate for 9 minutes at room temperature. Liquid was removed and samples were washed once with 1.5 mL PME buffer. 1.5 mL of ice-cold methanol was then added to each coverslip and samples were left to incubate for 10 minutes at room temperature. Samples were then washed twice with 1 mL PME buffer for 5 minutes. 2 mL of TBST (3% BSA) solution was added to each coverslip and samples were left to incubate for 30 minutes at room temperature.

200 μL of primary antibody solution (1:100 mouse α-HA) was added to the coverslips and samples were left to incubate for 1 hour at room temperature. Samples were then washed three times with 250 μL TBST for 5 minutes. 200 μL of secondary antibody solution (1:100 goat anti-mouse Alexa fluor 594-Abcam:ab150120) was added to each coverslip and samples were left to incubate at room temperature for 1 hour in the dark. Samples were washed with 250 μL TBST 3 times for 5 minutes. All liquid was removed and one drop of mounting medium (ProLong gold antifade mountant with DAPI-ThermoFisher Scientific:P36941) was added to a microscope slide. Each coverslip was placed germling side down on the microscope slide and the excess mounting medium was removed using filter paper. Nail polish was added to the microscope slides and was left to dry for 10 minutes at room temperature. Samples were stored overnight at 4 ^°^C in the dark. Localisations of proteins were detected using the Olympus FluoView1000 laser scanning confocal microscope.

**Protein domain and homology searches**

Detection of protein sizes and domains were performed using a combination of ScanProsite (de Castro et al., 2006) and InterPro softwares (Mitchell et al., 2019). Detection of protein homologs was performed by reciprocal BLAST searches (Altschul, Gish, Miller, Myers, & Lipman, 1990).

**Supplementary Figures**


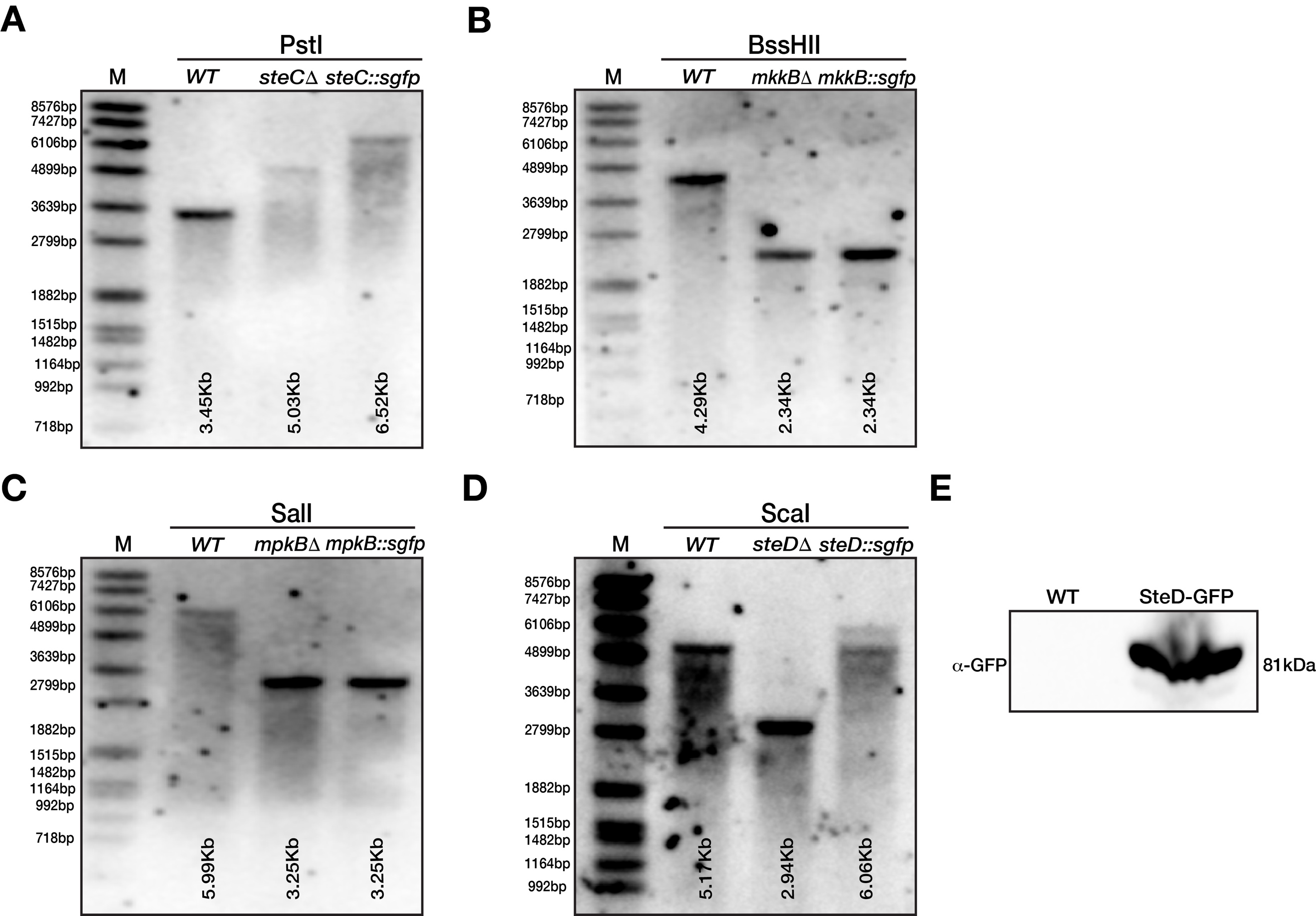


**Figure S1. Confirmation of deletions and tagged *A. flavus* strains via southern blotting.** (A) Southern hybridizations of *steC*Δ and *steC::sgfp*. M: Molecular marker in basepairs (bp). Sizes of the bands shown for the wild type TJES19.1 strain, the deletion strain and the tagged strain are in accordance with theoretical maps. The PstI restriction enzyme was used to digest genomic DNA and a 3’ UTR DIG-labelled probe was used for detection. (B) Southern hybridizations of *mkkB*Δ and *mkkB::sgfp*. The BssHII restriction enzyme was used to digest genomic DNA and a 3’ UTR DIG-labelled probe was used for detection. (C) Southern hybridizations of *mpkB*Δ and *mpkB::sgfp.* The SalI restriction enzyme was used to digest genomic DNA and a 3’ UTR DIG-labelled probe was used for detection. (D) Southern hybridizations of *steD*Δ and *steD::sgfp.* The faint upper band corresponds to the predicted size for *steD::sgfp* (6.06Kb). The ScaI restriction enzyme was used to digest genomic DNA and a 5’ UTR DIG-labelled probe was used for detection. (E) Western blot detecting the presence of the functional SteD-GFP fusion protein via an α-GFP antibody. The size of the tagged protein is 81kDa as predicted.


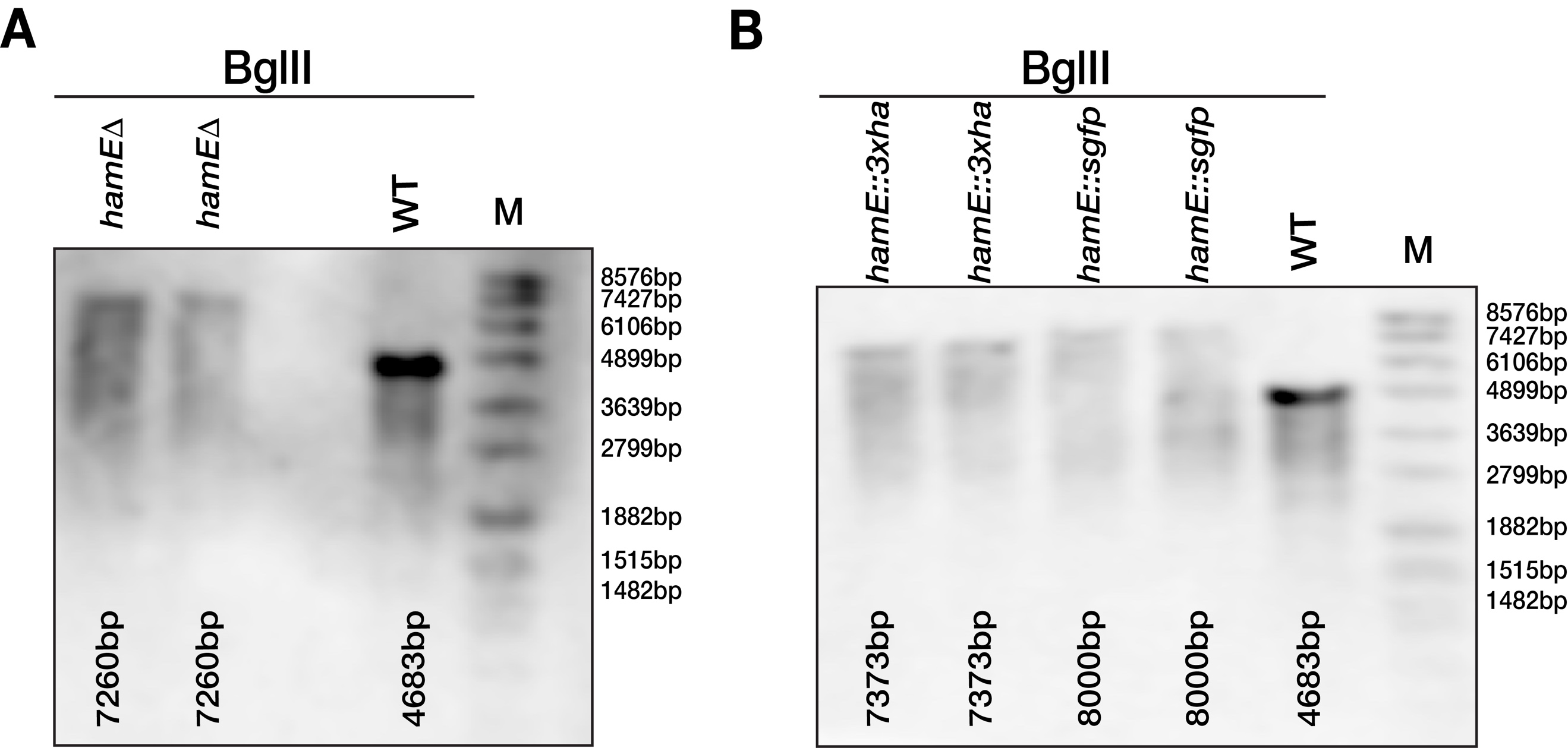


**Figure S2. Confirmation of *hamE* deletions and tagged *hamE* strains via southern blotting.** (A) Southern hybridizations of two *hamE*Δ clones. M: Molecular marker in basepairs (bp). Sizes of the bands shown for the wild type TJES19.1 strain, the deletion strain and the tagged strain are in accordance with theoretical maps. The BglII restriction enzyme was used to digest genomic DNA and a 3’ UTR DIG-labelled probe was used for detection. (B) Southern hybridizations of two *hamE::sgfp* and two *hamE::3xha* clones. The BglII restriction enzyme was used to digest genomic DNA and a 3’ UTR DIG-labelled probe was used for detection.

**
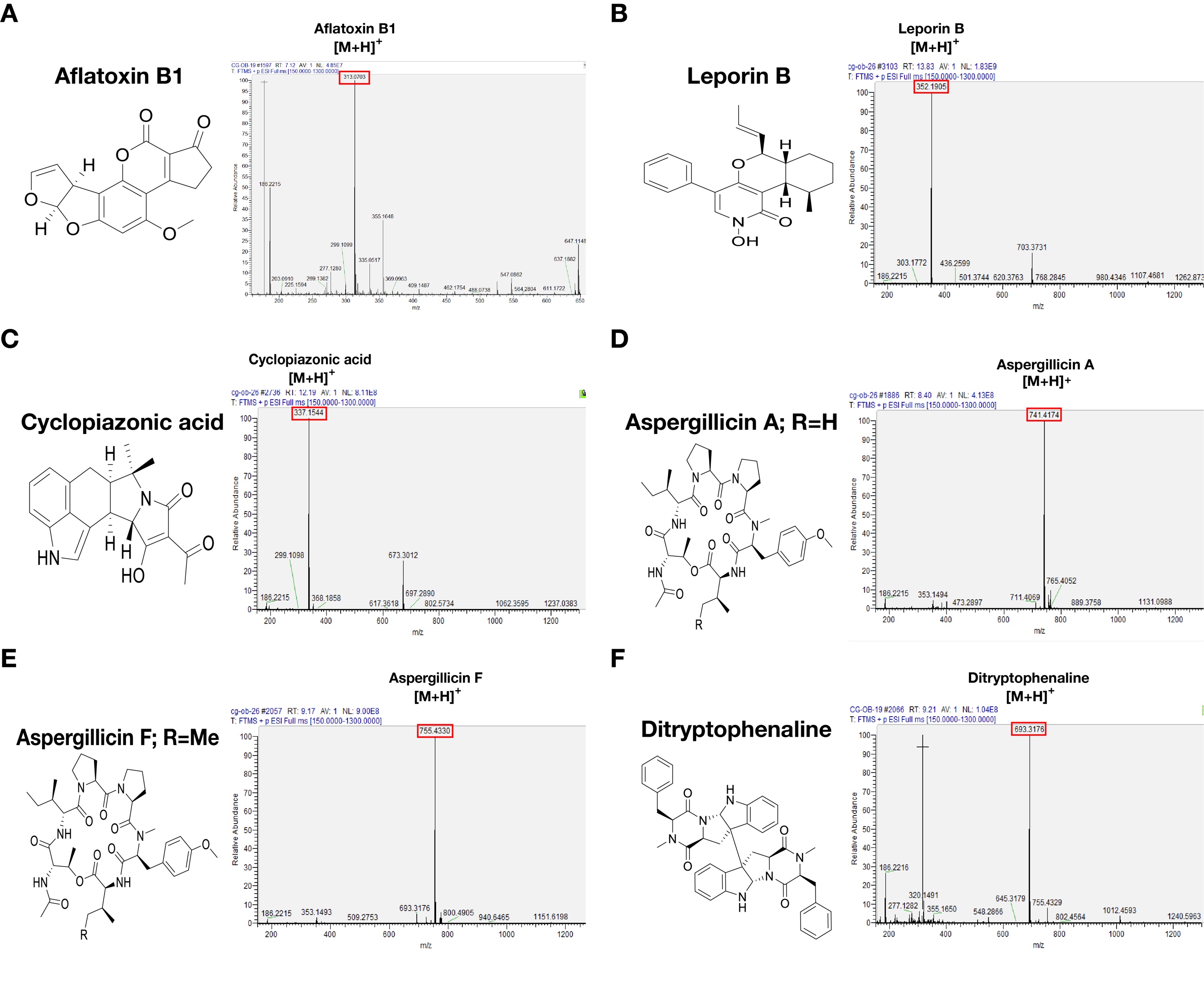
**

**Figure S3. HRMS spectra of various secondary metabolites detected by UHPLC-HRMS.** (A) Chemical structure and HRMS spectrum of aflatoxin B1. (B) Chemical structure and HRMS spectrum of leporin B. (C) Chemical structure and HRMS spectrum of cyclopiazonic acid. (D) Chemical structure and HRMS spectrum of aspergillicin A. (E) Chemical structure and HRMS spectrum of aspergillicin F. (F) Chemical structure and HRMS spectrum of ditryptophenaline.

**
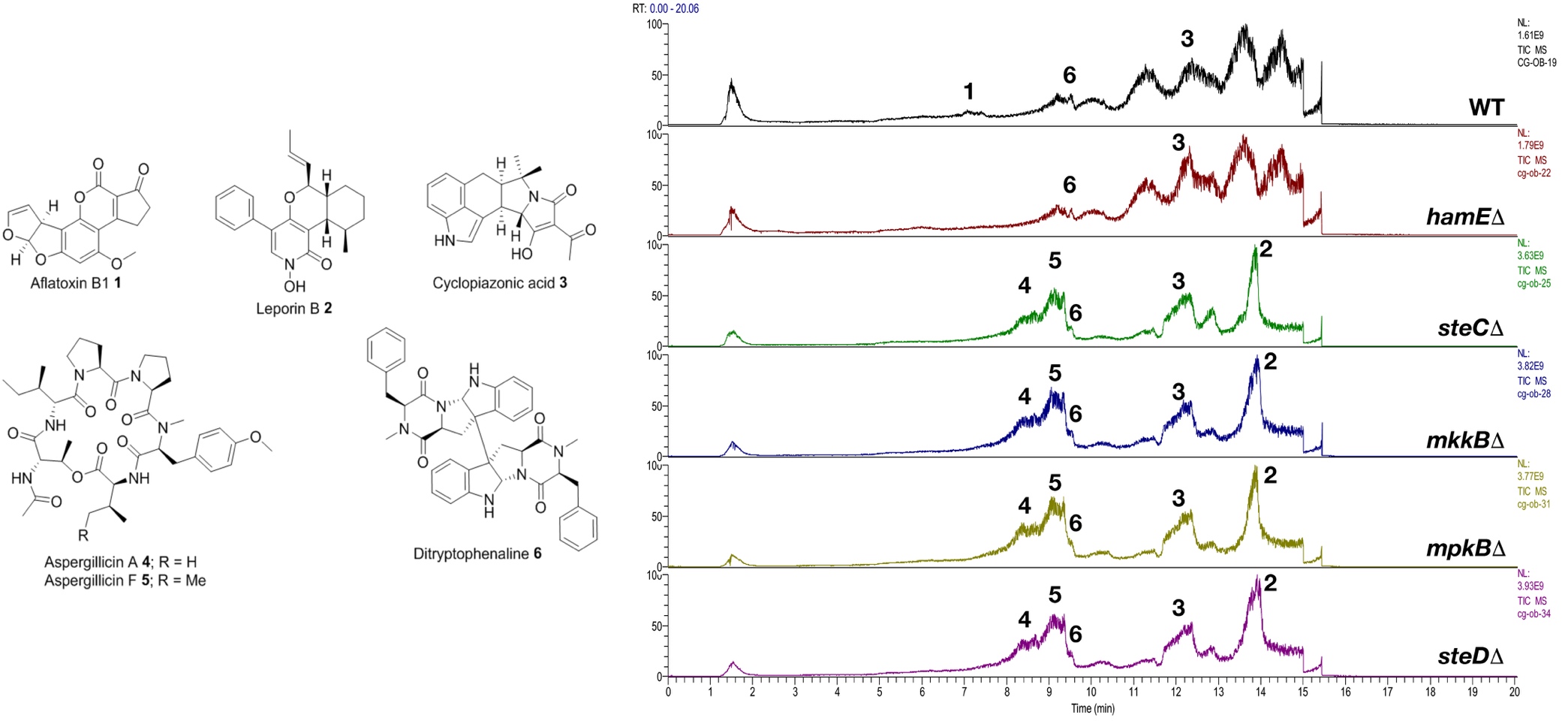
**

**Figure S4. UHPLC-HRMS total ion chromatograms of the TJES19.1 wild type strain and mutant strains.** Resulting spectra obtained following UHPLC-HRMS analysis of the TJES19.1 wild type strain and mutant strains cultured on PDA plates for 14 days at 30 ^°^C. Compounds detected are listed 1-6 and the respective chemical structures of each compound are presented.

**Supplementary Tables**

**Table S1:** Fungal strains created or used in this study

| **Strain** | **Genotype** | **Plasmid used** | **Reference** |
| --- | --- | --- | --- |
| TJES19.1 | *Wild type, nku70∆::argB, pyrG89* | Not applied | N. Keller Lab |
| AFLDF1 | *hamE∆::pyrG; nku70∆, pyrG89* | pDF1 in TJES19.1 | This Study |
| AFLDF2 | *hamE::sgfp::pyrG; nku70∆, pyrG89* | pDF2 in TJES19.1 | This Study |
| AFLDF3 | *hamE::3xha::pyrG; nku70∆, pyrG89* | pDF3 in TJES19.1 | This Study |
| AFLDF11 | *steC∆::phleO, nku70∆, pyrG89* | pDF30 in TJES19.1 | This Study |
| AFLDF12 | *mkkB∆::phleO, nku70∆, pyrG89* | pDF31 in TJES19.1 | This Study |
| AFLDF13 | *mpkB∆::phleO, nku70∆, pyrG89* | pDF32 in TJES19.1 | This Study |
| AFLDF14 | *steD∆::phleO, nku70∆, pyrG89* | pDF33 in TJES19.1 | This Study |
| AFLDF15 | *steC::sgfp::phleO, nku70∆, pyrG89* | pDF34 in TJES19.1 | This Study |
| AFLDF16 | *mkkB::sgfp::phleO, nku70∆, pyrG89* | pDF35 in TJES19.1 | This Study |
| AFLDF17 | *mpkB::sgfp::phleO, nku70∆, pyrG89* | pDF36 in TJES19.1 | This Study |
| AFLDF18 | *steD::sgfp::phleO, nku70∆, pyrG89* | pDF37 in TJES19.1 | This Study |
| AFLDF23 | *_p_hamE::hamE::hamE_t_, gpdA::phleO, hamE∆::pyrG, nku70∆, pyrG89* | pDF38 in AFLDF1 | This Study |
| AFLDF24 | *_p_steC::steC::steC_t_::pyrG, steC∆::phleO, nku70∆, pyrG89* | pDF39 in AFLDF11 | This Study |
| AFLDF25 | *_p_mkkB::mkkB::mkkB_t_::pyrG, mkkB∆::phleO, nku70∆, pyrG89* | pDF40 in AFLDF12 | This Study |
| AFLDF26 | *_p_mpkB::mpkB::mpkB_t_::pyrG, mpkB∆::phleO, nku70∆, pyrG89* | pDF41 in AFLDF13 | This Study |
| AFLDF27 | *_p_steD::steD::steD_t_::pyrG, steD∆::phleO, nku70∆, pyrG89* | pDF42 in AFLDF14 | This Study |

**Table S2:** Plasmids created or used in this study

| **Plasmid** | **Description** | **Reference** |
| --- | --- | --- |
| pUC19 | *E. coli* cloning plasmid with *bla* (ampicillin resistance) gene | Thermo Fisher |
| pAN8-1 | *gpdA::phleO* resistance cassette | This Study |
| pOSB113 | *Pme*I*::AfpyrG::Swa*I inserted in *Sma*I site of pUC19 | This Study |
| pDF1 | *hamE* deletion with *pyrG* in *Sma*I site of pUC19 | This Study |
| pDF2 | *hamE::sgfp::pyrG* cassette in *Sma*I site of pUC19 | This Study |
| pDF3 | *hamE::3xha::pyrG* cassette in *Sma*I site of pUC19 | This Study |
| pDF30 | *steC* deletion with *phleO* in *Sma*I site of pUC19 | This Study |
| pDF31 | *mkkB* deletion with *phleO* in *Sma*I site of pUC19 | This Study |
| pDF32 | *mpkB* deletion with *phleO* in *Sma*I site of pUC19 | This Study |
| pDF33 | *steD* deletion with *phleO* in *Sma*I site of pUC19 | This Study |
| pDF34 | *steC::sgfp::phleO* cassette in *Sma*I site of pUC19 | This Study |
| pDF35 | *mkkB::sgfp::phleO* cassette in *Sma*I site of pUC19 | This Study |
| pDF36 | *mpkB::sgfp::phleO* cassette in *Sma*I site of pUC19 | This Study |
| pDF37 | *steD::sgfp::phleO* cassette in *Sma*I site of pUC19 | This Study |
| pDF38 | *hamE* genomic locus in *Stu*I site of pAN8-1 | This Study |
| pDF39 | *steC* genomic locus in *Swa*I site of pOSB113 | This Study |
| pDF40 | *mkkB* genomic locus in *Swa*I site of pOSB113 | This Study |
| pDF41 | *mpkB* genomic locus in *Swa*I site of pOSB113 | This Study |
| pDF42 | *steD* genomic locus in *Swa*I site of pOSB113 | This Study |

**Table S3:** Oligonucleotides created or used in this study

| **Designation** | **Sequence in 5' > 3' direction** | **Size (basepairs)** |
| --- | --- | --- |
| DF01 (pDF1 5' UTR FWD, with pUC19 tail) | TTC GAG CTC GGT ACC CCT ACC GTT CCT TCT CCC TTC C | 37 |
| DF02 (pDF1 5' UTR REV, with *pyrG* tail) | GAG CAT TGT TTG AGG CGC TTC CGG TTG CAC CGG CA | 35 |
| DF03 (pDF1 3' UTR FWD, with *pyrG* tail) | GCC TCC TCT CAG ACA GCT ATT CGC CGA TCT TCG CTT TG | 38 |
| DF04 (pDF1 3' UTR REV, with pUC19 tail) | ACT CTA GAG GAT CCC CGA AGA GGT TCG CGG TTG CTG | 36 |
| DF05 (pDF1 5' FWD **nest oligo**) | CTC AAT CCG CCT CGT ACT AC | 20 |
| DF06 (pDF1 3' REV **nest oligo**) | CGA GTG TTT ATG CGG TCT ATA AAG | 24 |
| DF07 (pDF2/3 *hamE* ORF FWD with pUC19 tail) | TTC GAG CTC GGT ACC CGA ATA CCA TCT CCA GCG GCT G | 37 |
| DF08 (pDF2/3 ORF FWD **nest oligo**) | GAC CGA TCT GAC AGT CGC AAT G | 22 |
| DF09 (pDF2/3 *hamE* ORF REV with tail for GFP/HA linker) | CAC CGC TAC CAC CTC CGA TGC GAC CGT CGG CGA C | 34 |
| DF10 (pDF2/3 3' UTR FWD with tail for *pyrG*) | GCC TCC TCT CAG ACA GGT CAT TTT AAT TCT ATT CGC CGA TC | 41 |
| DF11 (pDF2/3 3' UTR REV **nest oligo**) | GGA GAT TAT ACA GGC CGC GAA G | 22 |
| DF12 (pDF2/3 3' UTR REV with pUC19 tail) | ACT CTA GAG GAT CCC CCA GGA AGT CGG AGT TGT ATC C | 37 |
| DF143 (pDF30 5' FWD with pUC19 tail) | TTC GAG CTC GGT ACC CCC ATC AAG AAG AAC GCC AGA C | 37 |
| DF144 (pDF30/34 3' REV with pUC19 tail) | ACT CTA GAG GAT CCC CCG ACC ACA TTG CTA TCC AGA TC | 38 |
| DF145 (pDF30 5' REV with *gpdA* promoter tail) | TTG ATG GTC GTT GTA GGG TTG AAA GGG GAA GCA ACC | 36 |
| DF146 (pDF30/34 3' FWD with *phleO* tail) | CGA GGA GCA GGA CTG AAA CCA GCC TGG TTG GAT GTG | 36 |
| DF147 (pDF30 5' FWD **nest oligo**) | GGA GCT AGC AGT TGT CAG C | 19 |
| DF148 (pDF30/34 3' REV **nest oligo**) | GAT CGT GGT CCT CTA CAC C | 19 |
| DF149 (pDF31 5' FWD with pUC19 tail) | TTC GAG CTC GGT ACC CCT ACG TCG CTT TCT CTT CTC C | 37 |
| DF150 (pDF31/35 3' REV with pUC19 tail) | ACT CTA GAG GAT CCC CCG TAT ATA GTG GTC CTC TGG TG | 38 |
| DF151 (pDF31 5' REV with *gpdA* promoter tail) | TTG ATG GTC GTT GTA GGG TTG GAC GGC AGA TTT ACT C | 37 |
| DF152 (pDF31/35 3' FWD with *phleO* tail) | CGA GGA GCA GGA CTG AGT AAA TAG AGT ACA TCA CTA TCG C | 40 |
| DF153 (pDF31 5' FWD **nest oligo**) | CCT CAT CGT CAT CAT CAT CAT C | 22 |
| DF154 (pDF31/35 3' REV **nest oligo**) | CGT TCG ATC CCA ACC ACT AAC | 21 |
| DF155 (pDF32 5' FWD with pUC19 tail) | TTC GAG CTC GGT ACC CGG ACC CTG AGA TTG CCT ATG | 36 |
| DF156 (pDF32/36 3' REV with pUC19 tail) | ACT CTA GAG GAT CCC CGG ACT CGA CAG AGC TAC TAC | 36 |
| DF157 (pDF32 5' REV with *gpdA* promoter tail) | TTG ATG GTC GTT GTA GAT TCG TGG ACT GTC CGA ACT AG | 38 |
| DF158 (pDF32/36 3' FWD with *phleO* tail) | CGA GGA GCA GGA CTG AGC TTG CAT TGG ACA GCT TGT C | 37 |
| DF159 (pDF32 5' FWD **nest oligo**) | GAG GTA CCT GAC TCC ATT ATG G | 22 |
| DF160 (pDF32/36 3' REV **nest oligo**) | CCT TCA ACA CCT CCT CGA CC | 20 |
| DF161 (pDF33 5' FWD with pUC19 tail) | TTC GAG CTC GGT ACC CGG TGG ATT CTG ACA GCG GAG | 36 |
| DF162 (pDF33/37 3' REV with pUC19 tail) | ACT CTA GAG GAT CCC CGA GCG TCG CAG TAC TCT CAA C | 37 |
| DF163 (pDF33 5' REV with *gpdA* promoter tail) | TTG ATG GTC GTT GTA GGG TGG TAG CGA ACA ATT TCT CG | 38 |
| DF164 (pDF33/37 3' FWD with *phleO* tail) | CGA GGA GCA GGA CTG ATC TGT CTG TTA CGG ACG TCG | 36 |
| DF165 (pDF33 5' FWD **nest oligo**) | GTG GGT ATT GAT TGG CGT TGG | 21 |
| DF166 (pDF33/37 3' REV **nest oligo**) | CTC CAC CTC AGC AAG ATG AC | 20 |
| DF167 (pDF34 5' FWD with pUC19 tail) | TTC GAG CTC GGT ACC CCC AAG CGA CGC ATA TCA ATG C | 37 |
| DF168 (pDF34 5' REV with tail for linker) | CAC CGC TAC CAC CTC CTG CAA TTG GCG TGG CGA GG | 35 |
| DF169 (pDF34 5' FWD **nest oligo**) | GTG CTG AAT ACG GAG CGA CAC | 21 |
| DF170 (pDF35 5' FWD with pUC19 tail) | TTC GAG CTC GGT ACC CAT GGC CGA CCA ATT CAA AGC TC | 38 |
| DF171 (pDF35 5' REV with tail for linker) | CAC CGC TAC CAC CTC CGC GTT GCC ATG GTG TTC C | 34 |
| DF172 (pDF35 5' FWD **nest oligo**) | CTG GAG ATC GGA CTG GAG TTC | 21 |
| DF173 (pDF36 5' FWD with pUC19 tail) | TTC GAG CTC GGT ACC CCT GTG GAT TCG CTC GAT GTT TG | 38 |
| DF174 (pDF36 5' REV with tail for linker) | CAC CGC TAC CAC CTC CCC GCA TGA TCT CCT CGT AG | 35 |
| DF175 (pDF36 5' FWD **nest oligo**) | CGA ATC GTT TGA CAG CAG ACA C | 22 |
| DF176 (pDF37 5' FWD with pUC19 tail) | TTC GAG CTC GGT ACC CGT CCA TGT GGC ACA CTC TC | 35 |
| DF177 (pDF37 5' REV with tail for linker) | CAC CGC TAC CAC CTC CTA GCA CTC CGC CGG GTA GAT TG | 38 |
| DF178 (pDF37 5' FWD **nest oligo**) | GTG TCG TGT CGG TCC TTA TC | 20 |
| DF247 (pDF38 5' forward primer with tail for pAN8-1) | CCC AAG ACC GAC AAG GAG GGC TGC AGT GTG AAG AAA C | 37 |
| DF248 (pDF38 3' reverse primer with tail for pAN8-1) | GCG TTC TGG AGG GAG GCG AAC TAG ATG CGA TGG TCA C | 37 |
| DF249 (pDF39 5' forward primer with tail for pOSB113) | AGC TCG GTA CCC ATT TGG ACG CAC CTC ATT ATG GAG | 36 |
| DF250 (pDF39 3' reverse primer with tail for pOSB113) | TTG AGG CGA ATT ATT TCC ATC TGA TCC CTC TTC CC | 35 |
| DF251 (pDF40 5' forward primer with tail for pOSB113) | AGC TCG GTA CCC ATT TCA GCA CCC TGA TGA GCT TC | 35 |
| DF252 (pDF40 3' reverse primer with tail for pOSB113) | TTG AGG CGA ATT ATT TCG ATC CCA ACC ACT AAC GC | 35 |
| DF253 (pDF41 5' forward primer with tail for pOSB113) | AGC TCG GTA CCC ATT TGT AGA TAC CGG ACC CTG AG | 35 |
| DF254 (pDF41 3' reverse primer with tail for pOSB113) | TTG AGG CGA ATT ATT TCG AGC TGG TTG ACC GTG AAA TC | 38 |
| DF255 (pDF42 5' forward primer with tail for pOSB113) | AGC TCG GTA CCC ATT TGT GGA TTC TGA CAG CGG AG | 35 |
| DF256 (pDF42 3' reverse primer with tail for pOSB113) | TTG AGG CGA ATT ATT TCT CTG AAG ACG ATG GCA CTG | 36 |
| DF302 (*veA* cDNA FWD) | CAA CCT CTC TCA ATC ATC CAG | 21 |
| DF303 (*veA* cDNA REV) | CTT CGT ACG ACC GCT TGG | 18 |
| DF304 (*velB* cDNA FWD) | GTT CAC GCG CAA CCT CAT C | 19 |
| DF305 (*velB* cDNA REV) | CCT TCA GTC CGT ACA CTC AG | 20 |
| DF308 (*wetA* cDNA FWD) | GCT CAC GCT AAA TAT GTT GAC G | 22 |
| DF309 (*wetA* cDNA REV) | GCA TGG CTA ATC CGT TCT TG | 20 |
| *aflA*-F (AFLA qPCR) (Chang, Scharfenstein, Mack, & Ehrlich, 2012) | CCT ATA AGT GCT TCA AAG ATC GTG ATC G | 28 |
| *aflA*-R (AFLA qPCR) (Chang et al., 2012) | CGT ACA TGG ATG ACA CGT TGT CCC AG | 26 |
| *aflC*-F (AFLA qPCR) (Chang et al., 2012) | CCT ATT CTA GCC GCC TTT CTT GAC | 24 |
| *aflC*-R (AFLA qPCR) (Chang et al., 2012) | CAT GTT GCC AGA TTC CTC ATA TTC C | 25 |
| *aflD*-F (AFLA qPCR) (Chang et al., 2012) | TGT ATG CTC CCG TCC TAC TGT TTC | 24 |
| *aflD*-R (AFLA qPCR) (Chang et al., 2012) | TGT AGT CTC CTT AGT CGC TTC ATC | 24 |
| *aflM*-F (AFLA qPCR) (Chang et al., 2012) | GCG GAG AAA GTG GTT GAA CAG ATC | 24 |
| *aflM*-R (AFLA qPCR) (Chang et al., 2012) | CAG CGA ACA AAG GTG TCA ATA GCC | 24 |
| *aflP*-F (AFLA qPCR) (Chang et al., 2012) | CGA TGT CTA TCT TCT CCG ATC TAT TC | 26 |
| *aflP*-R (AFLA qPCR) (Chang et al., 2012) | TCT CAG TCT CCA GTC TAT TAT CTA CC | 26 |
| *brlA*-F (AFLA qPCR) (Chang et al., 2012) | TAT CCA GAC ATT CAA GAC GCA CAG | 24 |
| *brlA*-R (AFLA qPCR) (Chang et al., 2012) | GAT AAT AGA GGG CAA GTT CTC CAA AG | 26 |
| *abaA*-F (AFLA qPCR) (Chang et al., 2012) | GAG TGG CAG ACC GAA TGT ATG TTG | 24 |
| *abaA*-R (AFLA qPCR) (Chang et al., 2012) | TAG TGG TAG GCA TTG GGT GAG TTG | 24 |
| BK276 (AFL *skpA* cDNA FWD) | CGA TGT TAG TCT TGC CTT GC | 20 |
| BK277 (AFL *skpA* cDNA RVS) | GAC CAG ATG AAA CTC AAG CTG | 21 |
| BK465 (AFLA *laeA* cDNA F) | CAC AAC TCT CGT GAT ACA ATC C | 22 |
| BK466 (AFLA *laeA* cDNA R) | GTA CCA GCG AGC AAC CTT TC | 20 |
| BK473 (AFLA *nsdD* cDNA F) | CAA TGT ACC AAG ACG AAT ACA AG | 23 |
| BK474 (AFLA *nsdD* cDNA R) | TGT CTC AGC TCG GTT ACA AC | 20 |
| BK587 (AFLA *flbB* cDNA F) | CTG ACA ACG CTG CTC AAC C | 19 |
| BK588 (AFLA *flbB* cDNA R) | CTT TAC GTC ATC TCT GGT CAA C | 22 |
| BK589 (AFLA *flbC* cDNA F) | CAT GAT GAG CCA GTT CAG TTC | 21 |
| BK590 (AFLA *flbC* cDNA R) | CAC CAG TGT GAC TGT ACA TG | 20 |
| BK591 (AFLA *flbD* cDNA F) | CCT AGG ACC GTC TCA TCG | 18 |
| BK592 (AFLA *flbD* cDNA F) | GTT GTC GGA CTT CTT CGA GC | 20 |
| BK609_AFCL4F | CAA GTC AGC ATG GTT GAC ATT C | 22 |
| BK610_AFCL4R | TCG TCG CAT CTT GTT CCG AG | 20 |
| BK617_AFCL8F | GTT GAT ATT CTG AAC CCA GAT G | 22 |
| BK618_AFCL8R | GGC AGC CAA CTC ATC AAG G | 19 |
| BK647_AFCL23F | CAG CGA GCG ATA TCT GGA G | 19 |
| BK648_AFCL23R | AGG ATC GCA TTC AAG GCA TC | 20 |
| BK709_AFCL54F | CGT CCT ACT TAA TCC CAC AC | 20 |
| BK710_AFCL54R | CTC GTC CAT GAC TGT ATC TG | 20 |

**Table S4:** SteC-GFP (CADAFLAP00010880) interacting proteins at 24 hours of vegetative growth. Proteins of interest are highlighted in yellow. SteD (CADAFLAP00010300), MpkB (CADAFLAP00002792).

**Table S4 (continued)**

**Table S5:** MkkB-GFP (CADAFLAP00012084) interacting proteins at 24 hours of vegetative growth. Proteins of interest are highlighted in yellow. SteC (CADAFLAP00010880), SteD (CADAFLAP00010300).

**Table S6:** MpkB-GFP (CADAFLAP00002792) interacting proteins at 24 hours of vegetative growth. Proteins of interest are highlighted in yellow. MkkB (CADAFLAP00012084), SteD (CADAFLAP00010300), SteA (CADAFLAP00010857).

**Table S7:** SteD-GFP (CADAFLAP00010300) interacting proteins at 24 hours of vegetative growth. Proteins of interest are highlighted in yellow. SteC (CADAFLAP00010880), MkkB (CADAFLAP00012084), MpkB (CADAFLAP00002792).

**Table S8:** HamE-HA (CADAFLAP00009262) interacting proteins at 24 hours of vegetative growth.

**Table S8 (continued)**

**Supplementary references**

Altschul, S. F., Gish, W., Miller, W., Myers, E. W., & Lipman, D. J. (1990). Basic local alignment search tool. *J Mol Biol, 215*(3), 403-410. doi:10.1016/s0022-2836(05)80360-2

Chang, P. K., Scharfenstein, L. L., Mack, B., & Ehrlich, K. C. (2012). Deletion of the *Aspergillus flavus* orthologue of *A. nidulans* fluG reduces conidiation and promotes production of sclerotia but does not abolish aflatoxin biosynthesis. *Appl Environ Microbiol, 78*(21), 7557-7563. doi:10.1128/aem.01241-12

de Castro, E., Sigrist, C. J., Gattiker, A., Bulliard, V., Langendijk-Genevaux, P. S., Gasteiger, E., . . . Hulo, N. (2006). ScanProsite: detection of PROSITE signature matches and ProRule-associated functional and structural residues in proteins. *Nucleic Acids Res, 34*, W362-365. doi:10.1093/nar/gkl124

Mitchell, A. L., Attwood, T. K., Babbitt, P. C., Blum, M., Bork, P., Bridge, A., . . . Finn, R. D. (2019). InterPro in 2019: improving coverage, classification and access to protein sequence annotations. *Nucleic Acids Res, 47*(D1), D351-d360. doi:10.1093/nar/gky1100
